# Structure of the microtubule-anchoring factor NEDD1 bound to the γ-tubulin ring complex

**DOI:** 10.1083/jcb.202410206

**Published:** 2025-05-21

**Authors:** Hugo Muñoz-Hernández, Yixin Xu, Aitor Pellicer Camardiel, Daniel Zhang, Allen Xue, Amol Aher, Ellie Walker, Florina Marxer, Tarun M. Kapoor, Michal Wieczorek

**Affiliations:** 1 Institute of Molecular Biology and Biophysics, ETH Zürich, Zürich, Switzerland; 2 Laboratory of Chemistry and Cell Biology, The Rockefeller University, New York, NY, USA

## Abstract

The γ-tubulin ring complex (γ-TuRC) is an essential multiprotein assembly that provides a template for microtubule nucleation. The γ-TuRC is recruited to microtubule-organizing centers (MTOCs) by the evolutionarily conserved attachment factor NEDD1. However, the structural basis of the NEDD1–γ-TuRC interaction is not known. Here, we report cryo-EM structures of NEDD1 bound to the human γ-TuRC in the absence or presence of the activating factor CDK5RAP2. We found that the C-terminus of NEDD1 forms a tetrameric α-helical assembly that contacts the lumen of the γ-TuRC cone and orients its microtubule-binding domain away from the complex. The structure of the γ-TuRC simultaneously bound to NEDD1 and CDK5RAP2 reveals that both factors can associate with the “open” conformation of the complex. Our results show that NEDD1 does not induce substantial conformational changes in the γ-TuRC but suggest that anchoring of γ-TuRC–capped microtubules by NEDD1 would be structurally compatible with the significant conformational changes experienced by the γ-TuRC during microtubule nucleation.

## Teaser

The γ-TuRC is recruited to microtubule-organizing centers by NEDD1. Muñoz-Hernández et al. report cryo-EM structures revealing how NEDD1’s C terminus forms an α-helical tetramer that docks onto the γ-TuRC without inducing major conformational changes in the complex, compatible with CDK5RAP2 binding and γ-tubulin ring closure.

## Introduction

Microtubules are nucleated by the γ-tubulin ring complex (γ-TuRC), a ∼2.3-MDa assembly that templates α/β-tubulin into a 13-protofilament, pseudo-helical microtubule lattice (Aher et al., 2024; Brito et al., 2024; Dendooven et al., 2024). The γ-TuRC is composed of γ-tubulin, GCP2, GCP3, GCP4, GCP5, GCP6, MZT1, and MZT2 (Kollman et al., 2011). The complex is an asymmetric cone with 14 “spokes,” each comprising a γ-tubulin molecule supported by a GCP2/3/4/5/6 subunit. The γ-TuRC’s asymmetry stems from the unique arrangement of GCP subunits, where: GCP2 and GCP3 alternate in positions 1–8; GCP4, GCP5, GCP4, and GCP6 occupy positions 9–12; and GCP2 and GCP3 fill positions 13 and 14 (Wieczorek et al., 2020b; Liu et al., 2020; Consolati et al., 2020; Zimmermann et al., 2020). The terminal GCP3 at position 14 overlaps with GCP2 at position 1, accommodating α-tubulin:β-tubulin contacts at the microtubule “seam” (Aher et al., 2024; Brito et al., 2024; Dendooven et al., 2024). GCP4-GCP6 may offer unique binding interfaces for γ-TuRC regulators (Wieczorek et al., 2020b), though specific partners remain unidentified.

Two well-documented factors that associate with the γ-TuRC to regulate its cellular functions are CDK5RAP2 (Choi et al., 2010), whose conserved CM1 motif induces conformational changes in the γ-TuRC that promote microtubule nucleation (Xu et al., 2024; Serna et al., 2024), and neural precursor cell expressed, developmentally downregulated 1 (NEDD1). NEDD1 is a cell cycle–regulated protein that is important for γ-TuRC localization and microtubule anchoring at centrosomes and within mitotic spindles (Lüders et al., 2006; Haren et al., 2006). NEDD1 upregulation is linked to solid tumor progression (Zhuo et al., 2024), suggesting therapeutic potential. NEDD1 is not essential for γ-TuRC assembly and has two key roles: recruiting the γ-TuRC to centrosomes in undifferentiated cells (Muroyama et al., 2016; Lüders et al., 2006; Haren et al., 2006) and promoting microtubule-mediated “branching” nucleation with the augmin complex in dividing cells (Liu and Wiese, 2008; Uehara et al., 2009; Goshima et al., 2008), neurons (Sánchez-Huertas et al., 2016; Cunha-Ferreira et al., 2018; Viais et al., 2021; Mukherjee et al., 2024; Zhang et al., 2024b), and plants (Hotta et al., 2012; Liu et al., 2014).

**Figure 1.**
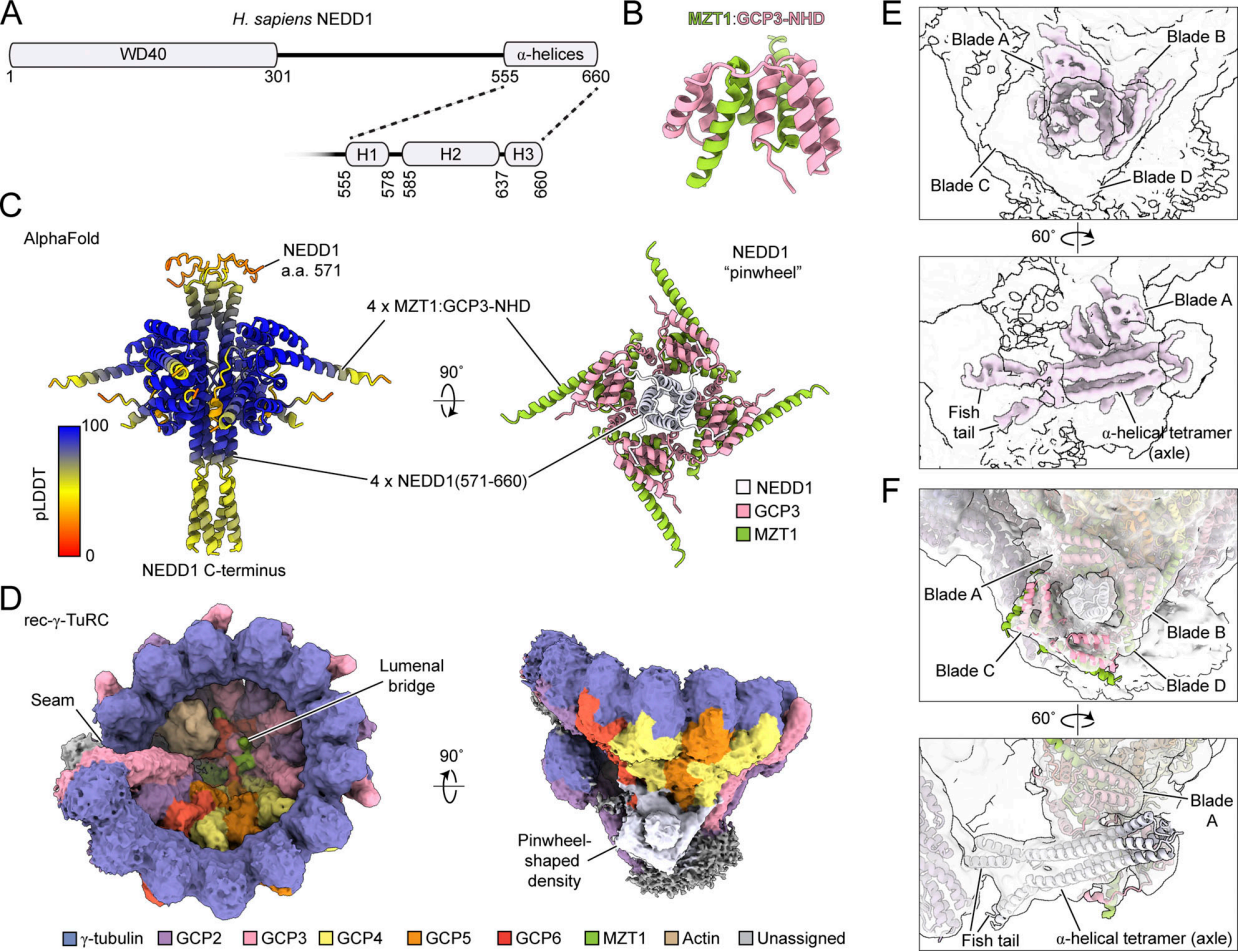
**A pinwheel-shaped structure consisting of a tetrameric NEDD1 helical bundle and four MZT1:GCP3-NHD modules docks onto the base of the asymmetric cone-shaped human γ-TuRC. (A)** Schematic of the human NEDD1 sequence. A zoom in on the C-terminal helical region predicted to form several α-helices is shown. Secondary structure predictions are taken from UniProt Consortium (2023). **(B)** Cartoon representation of the MZT1:GCP3-NHD structure in the γ-TuRC lumenal bridge, from PDB ID: 6X0U (Wieczorek et al., 2020a). **(C)** Two views of an AlphaFold prediction containing four copies each of NEDD1 residues 571–660, MZT1, and GCP3 residues 1–120. The model on the left is colored according to the predicted local distance difference test (pLDDT) score from the AlphaFold prediction. **(D)** Two views of the consensus rec-γ-TuRC density map (surface representation). The seam, lumenal bridge, and pinwheel-shaped densities are labeled; the pinwheel-shaped density is colored in lavender. Map resolution is 4.7 Å, but is shown at a low threshold to include features with weaker density. Higher resolution features can be found in Fig. S3 B. **(E)** Two views of the pinwheel density postprocessed using EMready (He et al., 2023) (light pink surface representation). The CryoSPARC postprocessed map is shown at the same threshold as in D as a transparent white surface for reference. The pinwheel axle containing the fishtail and α-helical tetramer, as well as pinwheel blades A–D, are indicated. **(F)** Two views of the refined γ-TuRC model in the rec-γ-TuRC consensus map, focusing on the pinwheel density. Blades C and D are omitted for clarity, and pinwheel features are labeled as in E.

NEDD1 consists of two structural domains: an N-terminal WD40 repeat domain (residues ∼1–301) and a C-terminal α-helical region (residues ∼555–660) (Fig. 1 A) (Manning et al., 2010). The WD40 domain is characterized by a β-propeller structure that targets NEDD1 to centrosomes (Haren et al., 2006), binds microtubules with weak affinity, and is essential for branching nucleation (Zhang et al., 2022). In contrast, the C-terminal region is α-helical (Fig. 1 A), forms tetramers in vitro (Manning et al., 2010), and recruits the γ-TuRC to centrosomes (Lüders et al., 2006; Haren et al., 2006). Despite NEDD1’s role in γ-TuRC function, how it interacts with the γ-TuRC is not clear. Moreover, while CDK5RAP2 transitions the γ-TuRC from an open, inactive state into a partially closed conformation with substantially improved microtubule-nucleating activity (Xu et al., 2024; Serna et al., 2024), it is unknown whether NEDD1 alters γ-TuRC conformation or accommodates the complex’s conformational changes during microtubule nucleation (Aher et al., 2024; Brito et al., 2024; Vermeulen et al., 2024).

It has been suggested that the C terminus of NEDD1 interacts with GCP3’s N-terminal α-helical domain (GCP3-NHD) (Zhang et al., 2022). GCP3-NHD is structurally homologous to the NHDs of GCP2, GCP5, and GCP6, which form subcomplexes with MZT1 (GCP3-, GCP5-, or GCP6-NHD) or MZT2 (GCP2-NHD). These MZT:GCP-NHD modules facilitate complex assembly and activation (Wieczorek et al., 2020a; Huang et al., 2020; Xu et al., 2024; Serna et al., 2024; Würtz et al., 2022) (Fig. 1 B). Notably, MZT1 has been proposed to mediate the γ-TuRC:NEDD1 interaction via GCP3’s N terminus (Cota et al., 2017; Zhang et al., 2022). Thus, evidence suggests that NEDD1 interacts with the γ-TuRC via MZT1:GCP3-NHD modules, but the structural details of these potential interactions remain unclear.

We report cryo-EM structures of NEDD1 bound to the human γ-TuRC. NEDD1’s C terminus forms a tetrameric α-helical bundle that interfaces with γ-TuRC components, including GCP4–6. Four MZT1:GCP3-NHD modules encircle the NEDD1 tetramer in a pinwheel-shaped structure. Mutations disrupting the pinwheel impaired NEDD1’s association with γ-tubulin complexes in cells. Our structures resolved missing features in the γ-TuRC, including MZT1:GCP5-NHD at the γ-TuRC seam that may be involved in early stages of microtubule nucleation (Brito et al., 2024). We also determined the structure of the γ-TuRC bound to both NEDD1 and an activating fragment of CDK5RAP2, showing that both factors can simultaneously bind to the “open” γ-TuRC conformation. Our results explain how NEDD1 binds the γ-TuRC and offer insights into the role of distinct regulatory protein-binding sites in γ-TuRC recruitment at different microtubule-organizing centers.

## Results and discussion

**Figure S1.**
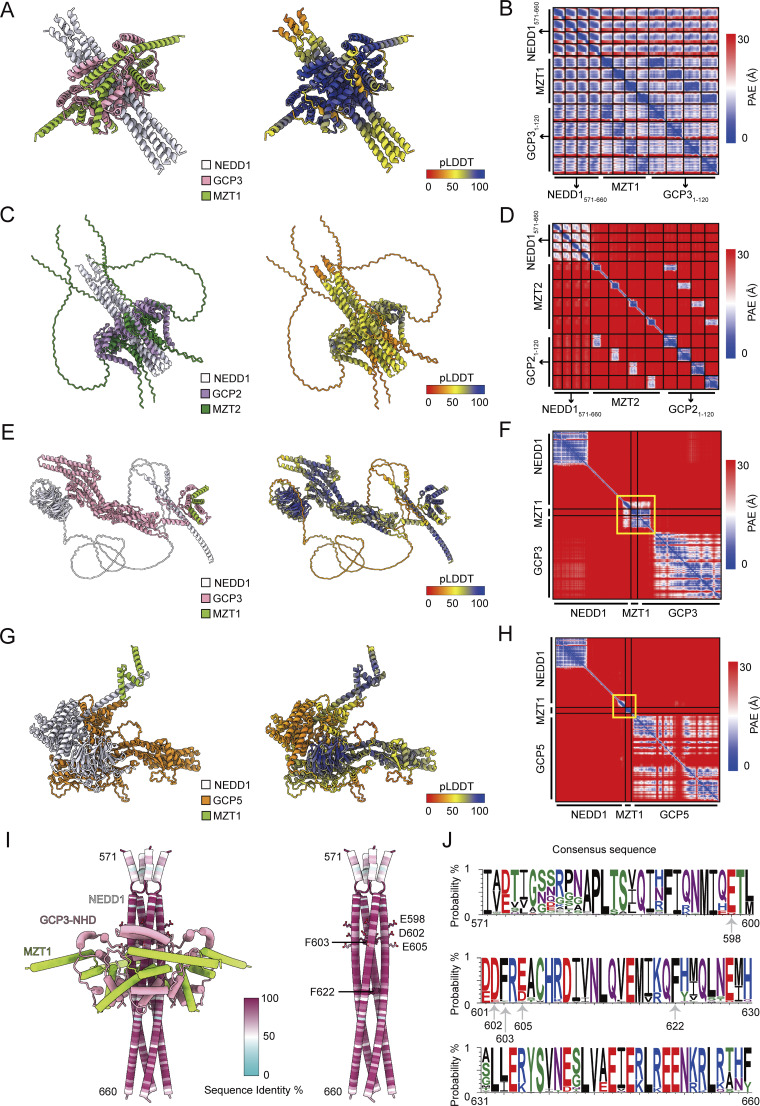
**AlphaFold predictions and NEDD1 conservation analysis. (A)** Cartoon representation of AlphaFold 3 prediction of four copies each of human NEDD1(571–660), MZT1, and GCP3(1–120) colored by subunit (left) and pLDDT (right). **(B)** Partial alignment error plot for the prediction in A. **(C)** Cartoon representation of AlphaFold 3 prediction of four copies each of human NEDD1(571–660), MZT2A, and GCP2(1–120) colored by subunit (left) and pLDDT (right). **(D)** Partial alignment error plot for the prediction in C. **(E)** Cartoon representation of AlphaFold 3 prediction of full-length *A. thaliana* NEDD1, MZT1A, and GCP3 colored by subunit (left) and pLDDT (right). **(F)** Partial alignment error plot for the prediction in E. **(G)** Cartoon representation of AlphaFold 3 prediction of full-length *A. thaliana* NEDD1, MZT1A, and GCP5A colored by subunit (left) and pLDDT (right). **(H)** Partial alignment error plot for the prediction in G. Yellow boxes in F and H highlight expected regions for the formation of NEDD1:MZT:GCP–NHD subcomplexes. **(I)** Cartoon representation of AlphaFold 3 model of the NEDD1 pinwheel in A. MZT1 and GCP3-NHD are colored according to the legend in A; NEDD1 is colored according to conservation as scored in the color key. A multiple sequence alignment of 141 annotated protein sequences across various species was used to score conservation. Residues mutated in this study are shown in stick representation on the right-hand model of NEDD1 alone. **(J)** Conservation of NEDD1 residues 571–660. Generated using WebLogo (Crooks et al., 2004).

To elucidate the structure of NEDD1, we used AlphaFold to predict subcomplexes of NEDD1 with MZT1:GCP3-NHD (Abramson et al., 2024). Four copies each of NEDD1 C-terminal residues 571–660, MZT1, and GCP3 residues 1–120 resulted in a high-confidence, “pinwheel”-shaped model, with four MZT1:GCP3-NHD modules encircling a NEDD1 tetramer (Fig. 1 C; and Fig. S1, A and B). The “axle” of the NEDD1 pinwheel comprises a four-helix bundle of NEDD1 α-helix H2 (Fig. 1 C), consistent with NEDD1 residues 572–660 forming a tetrameric α-helical assembly in vitro (Manning et al., 2010). Four MZT1:GCP3-NHD modules form the “blades” of the NEDD1 pinwheel (Fig. 1 C), consistent with reported interactions between MZT1, GCP3-NHD, and NEDD1 (Cota et al., 2017; Zhang et al., 2022). For comparison, an AlphaFold prediction for four copies each of MZT2:GCP2-NHD and NEDD1 had significantly lower confidence scores (Fig. S1, C and D). These observations suggest that NEDD1 and MZT1:GCP3-NHD form a hetero-dodecameric subcomplex that may be associated with the γ-TuRC.

To test this hypothesis, we determined the cryo-EM structure of a NEDD1-containing, reconstituted human γ-TuRC (“rec-γ-TuRC”) (Wieczorek et al., 2021). Previous low- to medium-resolution 3D reconstructions of rec-γ-TuRC consistently showed unassigned densities at the bottom of the cone-shaped assembly (Wieczorek et al., 2021; Aher et al., 2024) (Fig. 1 D). These densities are absent in other γ-TuRC structures using complexes with little or no NEDD1 (Wieczorek et al., 2020b; Liu et al., 2020; Zimmermann et al., 2020; Consolati et al., 2020; Xu et al., 2024; Würtz et al., 2022; Dendooven et al., 2024; Vermeulen et al., 2024), making them reasonable candidates for NEDD1 assignment.

**Figure S2.**
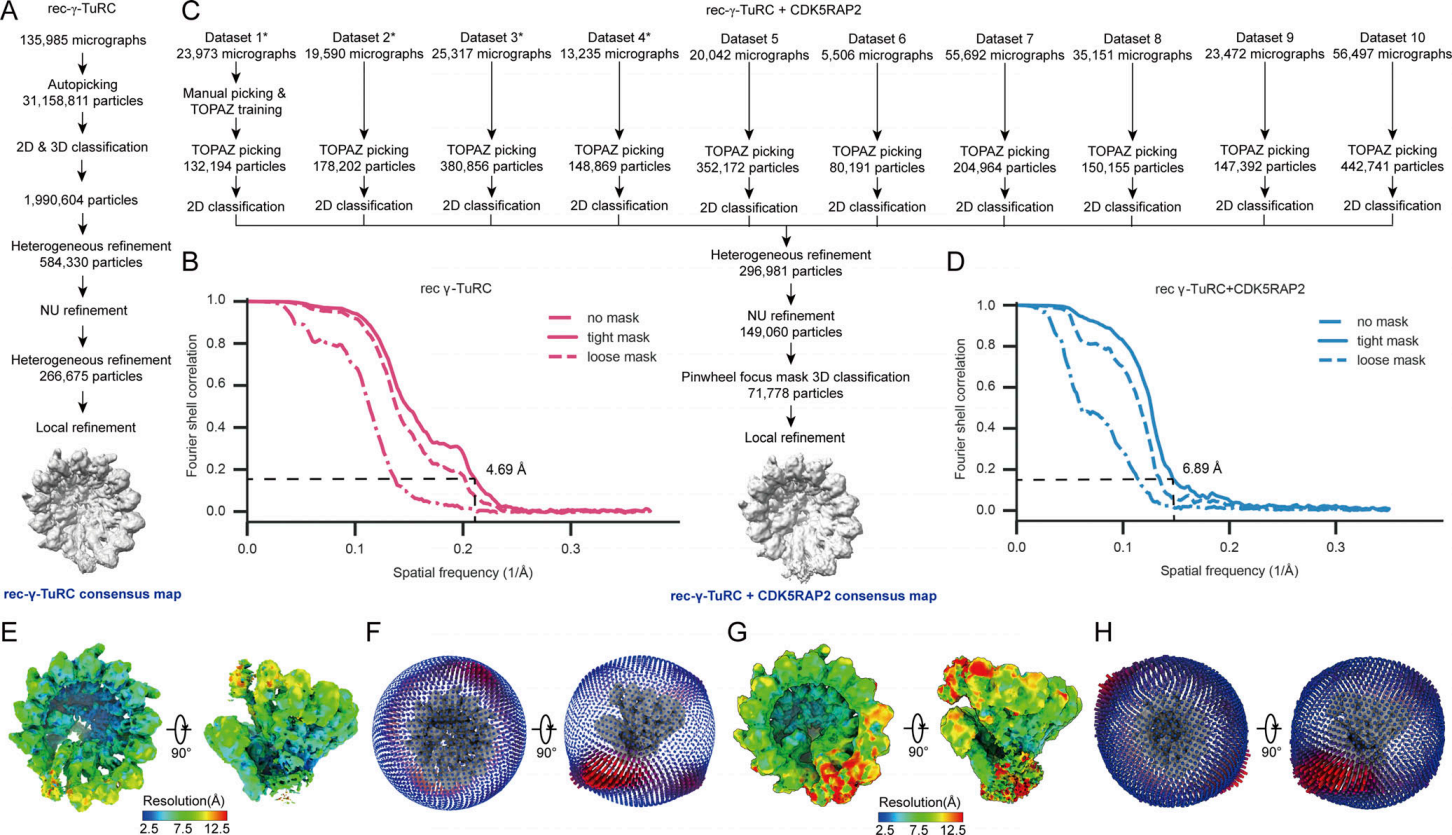
**Cryo-EM processing pipeline. (A)** Summary of the new rec-γ-TuRC cryo-EM data processing strategy. Cryo-EM collection details were reported in Aher et al. (2024). **(B)** Gold-standard Fourier shell correlation (FSC) plot for the consensus rec-γ-TuRC density map. The FSC at 0.143 is indicated by a dashed line. **(C)** Summary of new rec-γ-TuRC + CDK5RAP2 cryo-EM data processing strategy. Cryo-EM collection details for datasets 1–4 (marked by asterisks) were reported in Xu et al. (2024). **(D)** Gold-standard FSC plot for the consensus rec-γ-TuRC + CDK5RAP2 density map. The FSC at 0.143 is indicated by a dashed line. **(E)** Two views of the consensus rec-γ-TuRC density map analyzed by CryoSPARC, showing a resolution distribution ranging from 2.5 to >12.5 Å. **(F)** Two views of the particle angular distribution overlaid onto the rec-γ-TuRC consensus map. **(G)** Two views of the consensus rec-γ-TuRC + CDK5RAP2 density map analyzed by CryoSPARC, showing a resolution distribution ranging from 2.5 to >12.5 Å. **(H)** Two views of the particle angular distribution overlaid onto the rec-γ-TuRC + CDK5RAP2 consensus map.

**Figure S3.**
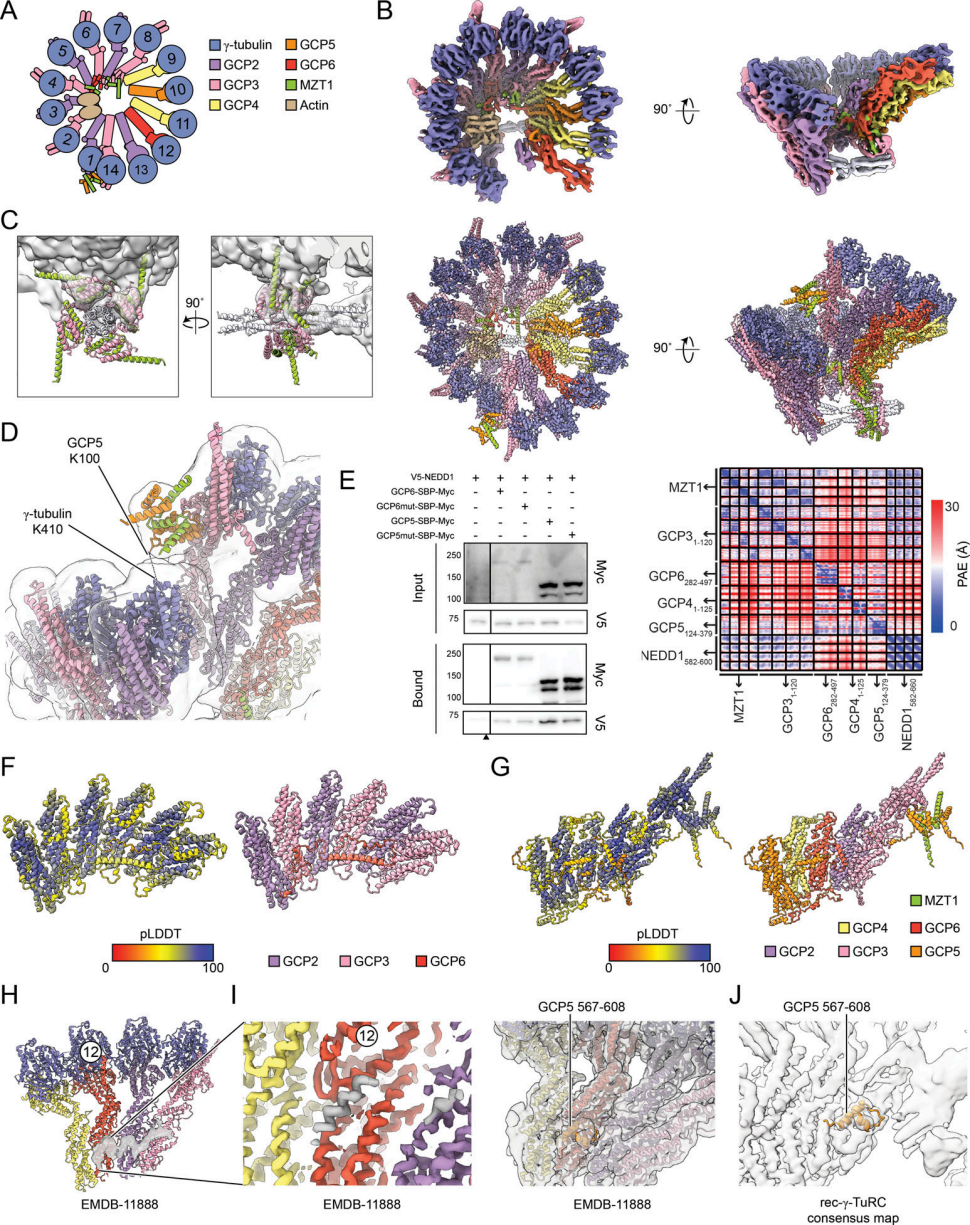
**Details regarding rec-γ-TuRC consensus reconstruction and model building. (A)** Schematic of the γ-TuRC highlighting subunit composition and numbering across the complex. **(B)** Top: two views of the rec-γ-TuRC consensus map showing higher resolution features. Map was sharpened in CryoSPARC and postprocessed with EMready (He et al., 2023). Bottom: two views of the refined rec-γ-TuRC model, including the NEDD1 pinwheel. **(C)** Two views of the NEDD1 pinwheel predicted by AlphaFold 3 (cartoon representation) fitted into the pinwheel density in the rec-γ-TuRC consensus map (transparent surface). **(D)** Cartoon representation view of MZT1:GCP5-NHD at the rec-γ-TuRC seam with the consensus density map in transparent surface representation. GCP5 K100 and γ-tubulin K410, identified as cross-linked residues in the native human γ-TuRC, are indicated (Consolati et al., 2020). **(E)** Left: western blot of inputs and bound fractions of SBP pulldowns of GCP-SBP-Myc constructs from HEK293T cells. GCP6mut corresponds to a deletion of GCP6 residues 329–341, while GCP5mut corresponds to a quadruple mutant of GCP5 R213A/R228G/L256E/V258E. Cells untransfected with any GCP-SBP-Myc constructs served as a negative control. Black triangle indicates location where blots were cropped for final figure generation. The experiment was performed three times with similar results. Right: partial alignment error plot for the AlphaFold prediction in Fig. 2 I. **(F)** Cartoon representation of AlphaFold 3 prediction of three copies each of GCP2 and GCP3 GRIP1 domains, together with the GCP6 belt and residues 191–252, colored by pLDDT (left) and subunit (right). **(G)** Cartoon representation of AlphaFold 3 prediction of GRIP1 domains of GCP4, GCP5 (including NHD), GCP6, and GCP2, as well as MZT1 and the GRIP1 and GRIP2 domains of GCP3, colored by pLDDT (left) and subunit (right). The GCP5 insertion element that contacts the lumenal face of GCP6 is indicated. **(H and I)** Segmented surface representation of the previously described helical element lining the lumenal face of GCP6, 2, and 3 in EMDB-11888 (Zimmermann et al., 2020). Map was postprocessed with EMready (He et al., 2023). The γ-TuRC subunits from the same study are shown in cartoon representation for reference. A zoomed in view of an unassigned helix contacting GCP6 is shown in I (left) and at a higher threshold. The right shows the GCP5 insertion (aa 567–608) modeled in this study in cartoon representation and fitted into the EMDB-11888 density map (Zimmermann et al., 2020). **(J)** The GCP5 insertion modeled in this study (aa 567–608) is shown in cartoon representation in the rec-γ-TuRC density map. γ-TuRC position 12 corresponding to GCP6 is indicated in panels G, I, and J for reference. pLDDT, predicted local distance difference test. Source data are available for this figure: SourceData FS3.

To resolve these unassigned densities, we analyzed “free” rec-γ-TuRC particles in micrographs from a recent cryo-EM study of rec-γ-TuRC–capped microtubule ends (Aher et al., 2024). In a new processing pipeline, we re-picked particles, cleaned them in RELION (Kimanius et al., 2021), and performed supervised 3D classifications and refinements in CryoSPARC (Punjani et al., 2017). This improved the rec-γ-TuRC resolution from ∼7 to 4.7 Å (Fig. 1 D; Fig. S2, A, B, E, and F; Fig. S3, A and B; and Table S2) and resolved unassigned densities associated with GCP4–6 at γ-TuRC positions 9–12, which, at low thresholds, resemble the AlphaFold-predicted NEDD1 pinwheel model (Fig. 1 D). The NEDD1 pinwheel could be rigid body fitted into this density (Fig. S3 C), indicating that NEDD1 binds to the bottom of the γ-TuRC.

The fitted AlphaFold model agreed particularly well with densities corresponding to the axle and blades A and B (Fig. S3 C). Densities for blades C and D agreed with the fitted model at low thresholds but had overall weaker density, complicating secondary structure assessments. MZT1:GCP5-NHD, MZT1:GCP6-NHD, and MZT2:GCP2-NHD all have qualitatively similar folds as MZT1:GCP3-NHD (Wieczorek et al., 2020a; Huang et al., 2020) and could theoretically constitute one or more of the blade densities. However, the following evidence support the exclusive assignment of MZT1:GCP3-NHD to the NEDD1 pinwheel: (1) GCP6-NHD is already assigned to the lumenal bridge as an actin-binding domain (Fig. 1 D) (Wieczorek et al., 2020a, 2021; Liu et al., 2020; Consolati et al., 2020; Zimmermann et al., 2020; Xu et al., 2024; Würtz et al., 2022); (2) MZT2 is absent in NEDD1-containing organisms like *Drosophila melanogaster* (Tovey and Conduit, 2018), and MZT2:GCP2-NHD is not predicted to form a complex with NEDD1 (Fig. S1, C and D); (3) NEDD1 is conserved in flowering plants (Zeng et al., 2009), and *Arabidopsis thaliana* NEDD1 is predicted to interact with MZT1:GCP3-NHD (Fig. S1, E and F), but not with MZT1 and the N-terminal portion of GCP5 (Fig. S1, G and H); and (4) a deep learning framework for identifying human protein–protein interactions ranked GCP3 as NEDD1’s top binding partner (Zhang et al., 2024a, *Preprint*). Thus, the pinwheel density likely contains four copies each of NEDD1, MZT1, and GCP3-NHD.

### The NEDD1 pinwheel associates with the γ-TuRC through conserved interfaces

**Figure 2.**
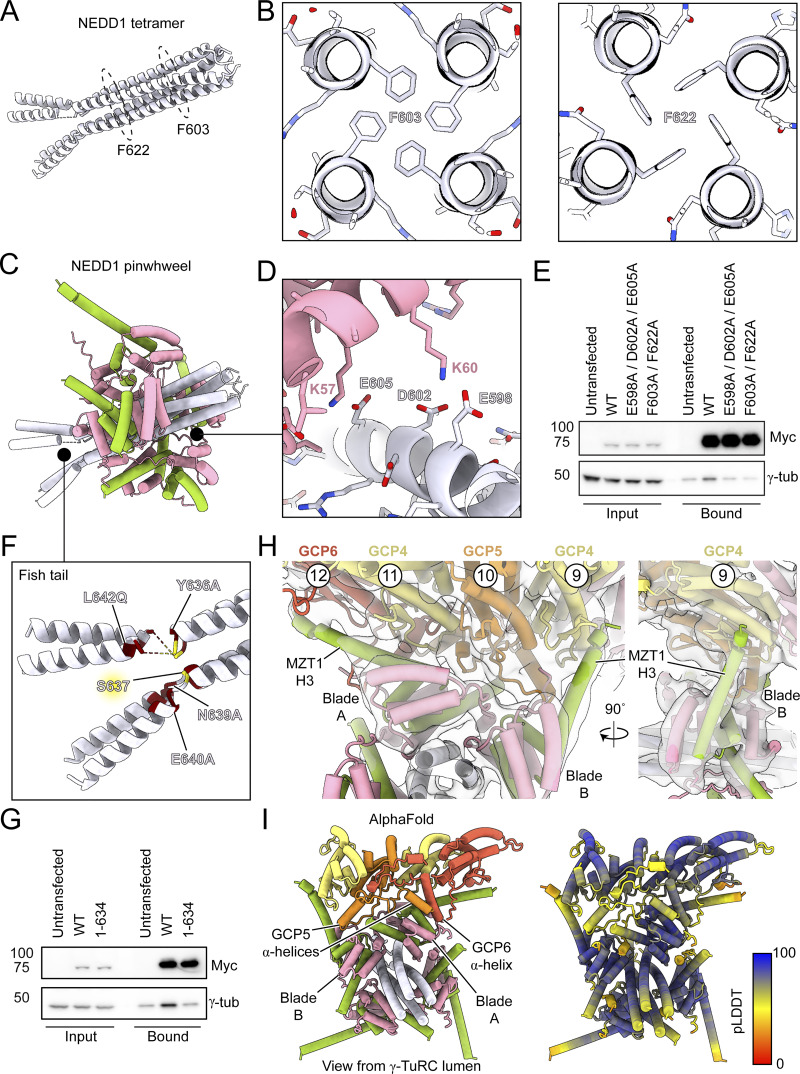
**The NEDD1 pinwheel associates with the γ-TuRC through multiple interfaces. (A)** Cartoon representation of a model of the NEDD1 C-terminal tetramer. Locations of conserved F603 and F622 residues are highlighted by dashed circles. **(B)** Cross-section views of the NEDD1 tetramer AlphaFold model showing the predicted packing of F603 (left) and F622 (right). **(C)** Cartoon representation of a model of the NEDD1 pinwheel, color as in Fig. 1 F. Black circles indicate zoom in areas of interest for panels D and F. **(D)** Zoom in view of NEDD1 pinwheel AlphaFold model for regions specified in C, showing conserved residues involved in electrostatic interactions between NEDD1 and GCP3 in the pinwheel. **(E)** Western blot of inputs and bound fractions of SBP pulldowns of Myc-SBP-NEDD1 constructs from HEK293T cells. Untransfected cells served as a negative control. **(F)** Zoom in view of the fishtail region of the NEDD1 pinwheel model, highlighting previously reported mutations that abolish NEDD1:γ-TuRC interactions (maroon) (Manning et al., 2010), as well as an identified Plk1 phosphorylation site (yellow) (Zhang et al., 2009). **(G)** Western blot of inputs and bound fractions of SBP pulldowns of Myc-SBP-NEDD1 constructs from HEK293T cells. 1–634 refers to a NEDD1 fishtail deletion lacking residues 635–660. Untransfected cells served as a negative control. **(H)** Two views of the NEDD1 pinwheel Blades A and B bound to the base of the γ-TuRC (cartoon representation with cryo-EM density in transparent grey surface). **(I)** Lumenal view of an AlphaFold prediction of the NEDD1 pinwheel contacting the GRIP1 domains of GCP4–6. The model on the right is colored according to the pLDDT. pLDDT, predicted local distance difference test. Experiments in E and G were performed three times with similar results. Source data are available for this figure: SourceData F2.

We next used our density maps and AlphaFold predictions to build a molecular model of the human γ-TuRC bound to the NEDD1 pinwheel (Fig. 1, E and F; Fig. S3 B; and Tables S2 and S3). The refined NEDD1 pinwheel model revealed conserved interfaces with MZT1:GCP3-NHD modules (Fig. S1, I and J). NEDD1 residues F603 and F622 form hydrophobic cores that may be critical for NEDD1 helical assembly (Fig. 2, A and B), and electrostatic interactions between NEDD1 residues E598, D602, and E605 and GCP3-NHD residues K57 and K60 likely stabilize the NEDD1-GCP3-NHD interface (Fig. 2, C and D). Mutating either of these residue sets to alanine (F603A/F622A and E598A/D602A/E605A) both reduced the ability of overexpressed NEDD1 to pull down γ-tubulin from HEK293T cells (Fig. 2 E). These results validate the γ-TuRC-NEDD1 pinwheel model and identify two critical interfaces for NEDD1–γ-TuRC interactions.

We next analyzed the interaction between the NEDD1 pinwheel and the γ-TuRC, which is mediated by two main interfaces. In the first interface, blades A and B of the NEDD1 pinwheel contact the underside of the γ-TuRC (Fig. 2 H). Blade B lies beneath GCP4’s GRIP1 domain at position 9, while blade A interfaces with the GRIP1 domains of GCP5, GCP4, and GCP6 at positions 10–12 (Fig. 2 H). In blade B, MZT1’s C-terminal α-helix (H3) extends along GCP4’s GRIP1 domain (Fig. 2 H). A GCP5 α-helical element (residues ∼243–263) inserts into a pocket in blade B lined by MZT1 α-helices H2 and H3 (Fig. 2, H and I). In blade A, MZT1 α-helix H3 spans the lower part of GCP4’s and GCP6’s GRIP1 domains, with α-helices formed by GCP5 residues ∼210–220 and GCP6 residues ∼325–343 inserting into a pocket in blade B lined by MZT1 H2 and H3. Mutagenesis of GCP5 hydrophobic residues or partial deletion of the GCP6 α-helix did not reduce NEDD1 levels detected in pull-downs from cultured cells (Fig. S3 E), suggesting redundant binding elements in this large (greater-than ∼1,500 Å^2^) interface. These findings clarify how the pinwheel blades bind to the γ-TuRC, with GCP5 and GCP6-specific elements aiding pinwheel orientation, alongside other interfaces (Würtz et al., 2022; Zimmermann et al., 2020).

**Figure 3.**
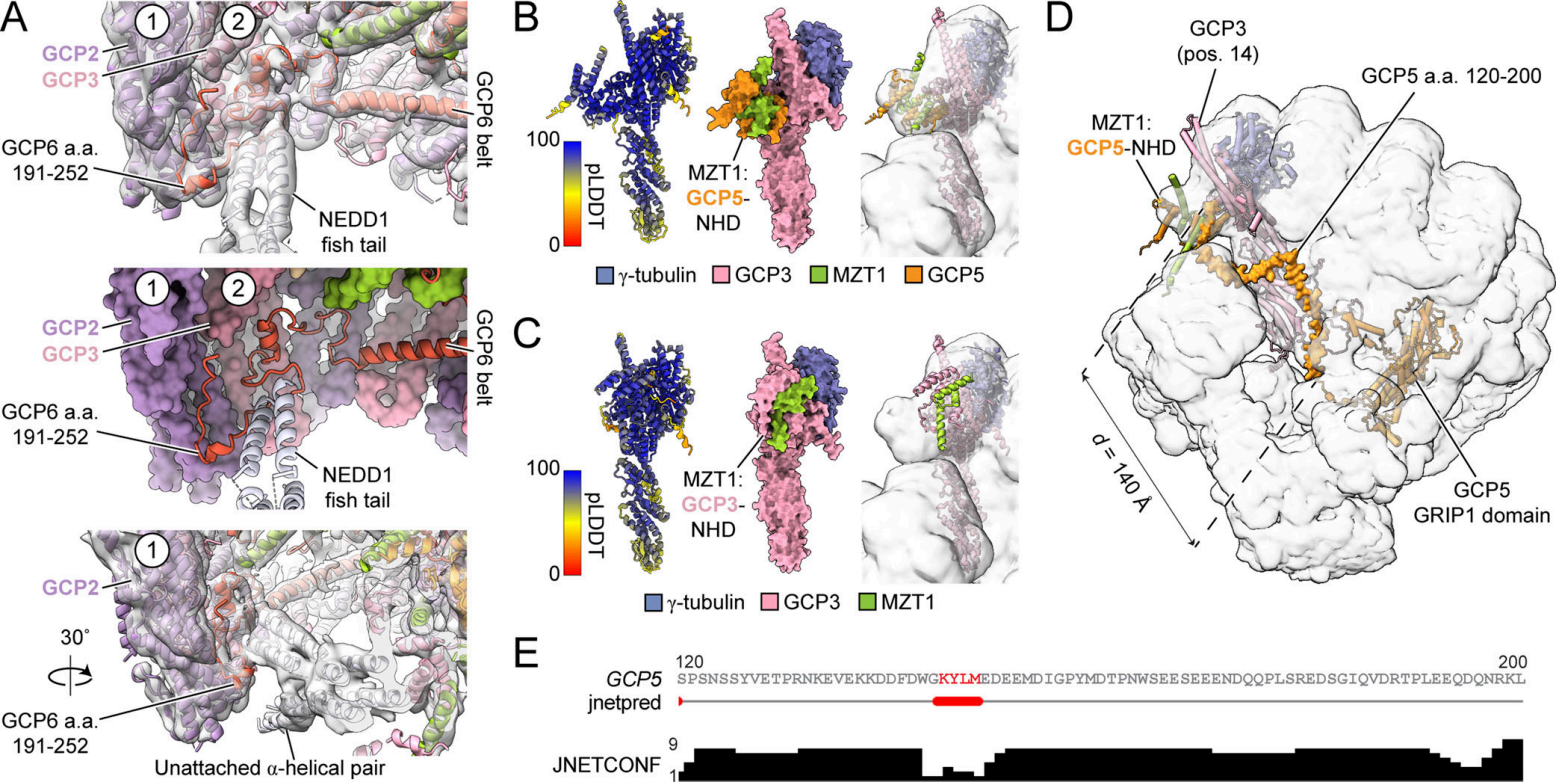
**The NEDD1 pinwheel contacts an extension in the GCP6 belt and enables the assignment of MZT1:GCP5-NHD to the γ-TuRC seam. (A)** Two views of the upper helical pair of the NEDD1 fishtail contacting positions 1 and 2 of the γ-TuRC (top: cartoon representation and cryo-EM density in transparent grey surface; middle: cartoon representation of NEDD1 and GCP6 next to a surface representation of the γ-TuRC). Newly modeled GCP6 residues 191–252 extending from the GCP6 belt are indicated. The bottom panel shows a rotated view of the same interface to highlight the unattached helical pair in the fishtail. γ-TuRC subunit positions 1 (GCP2) and 2 (GCP3) are indicated, where possible. **(B)** AlphaFold model of GCP3, MZT1:GCP5-NHD, and γ-tubulin rigid-body fitted in the rec-γ-TuRC consensus map (transparent representation; right). The model on the left is colored according to the pLDDT score from the AlphaFold prediction (cartoon representation). The model in the middle is shown in surface representation and is colored according to the legend. **(C)** AlphaFold model of GCP3, MZT1:GCP3-NHD, and γ-tubulin rigid-body fitted in the rec-γ-TuRC consensus map (transparent representation; right). The model on the left is colored according to the pLDDT score from the AlphaFold prediction (cartoon representation). The model in the middle is shown in surface representation and is colored according to the legend. **(D)** Rigid body-fitted AlphaFold model of GCP5, GCP4, GCP6, GCP2, GCP3, and MZT1 in the rec-γ-TuRC consensus map (transparent representation). MZT1:GCP5-NHD, GCP3, and the disordered GCP5 linker (aa 120–200) are indicated. The Euclidean distance between GCP5 residues 120 and 200 in the model is indicated. **(E)** Secondary structure prediction of human GCP5 residues 120–200. Top: primary sequence (red = predicted α-helices); middle: jnetpred secondary structure prediction result (red = α-helices); bottom: confidence score for the prediction. Figure panel generated using Jalview (Waterhouse et al., 2009). pLDDT, predicted local distance difference test.

In the second NEDD1 pinwheel–γ-TuRC interface, the C terminus of the NEDD1 tetrameric α-helical bundle density (α-helices H2; Fig. 1 A) splays apart into two helical pairs (α-helices H3), forming a “fishtail” (Fig. 1, E and F; and Fig. 2 F). The loop between H2 and H3 contains previously reported mutations in NEDD1 that disrupted its ability to co-immunoprecipitate γ-tubulin (Manning et al., 2010) (Fig. 2 F). The fishtail’s upper H3 pair contacts GRIP1 domains of GCP2 (position 1) and GCP3 (position 2), while the lower pair is unattached and less well-resolved (Fig. 3 A and Fig. S3 B). Deleting NEDD1’s fishtail region, including previously reported mutation sites (Manning et al., 2010), reduced γ-tubulin binding in cells (Fig. 2 G), suggesting that it forms an important binding interface. The fishtail:GCP2/3 connection interaction is mediated by a loop extending from the GCP6 “belt” (Wieczorek et al., 2020a) (Fig. 3 A; top). An AlphaFold 3 prediction of GCP6 belt-adjacent residues 191–252 with the GRIP1 domains of GCP2 and GCP3 matched our density (Fig. 3 A and Fig. S3 F), arguing that the GCP6 belt extends along the lumenal face of GCP2 and GCP3 to connect with the NEDD1 fishtail (Fig. 3 A). These observations refine existing models of GCP6 and reveal that NEDD1 binds to the γ-TuRC directly via the fishtail, as well as indirectly via two MZT1:GCP3-NHD modules.

### The structure of the NEDD1-bound γ-TuRC enables assignment of GCP5-specific features

We identified an unassigned α-helical element along the lumenal face of GCP6’s GRIP1 domain (Fig. S3 H), which was noted in previous γ-TuRC reconstructions (Zimmermann et al., 2020; Xu et al., 2024). AlphaFold predicted that an insertion element in GCP5 corresponding to residues ∼567–608 form a helix-turn-helix motif that contacts a pocket in GCP6’s GRIP1 domain (Fig. S3 G). The model fits well into previous γ-TuRC reconstructions (Zimmermann et al., 2020) and our own (Fig. S3, I and J). GCP5’s insertion loops back toward GCP5, indicating that the remaining densities crossing toward GCP3 and GCP2 at positions 13–14 must constitute a separate polypeptide chain(s) (Zimmermann et al., 2020), though its identity remains unclear due to resolution limits.

The γ-TuRC features a MZT:GCP-NHD module-shaped density next to GCP3’s GRIP2 domain, positioned above γ-tubulin at position 1 at the seam (Fig. 1 D). This module may “cap” γ-TuRC assembly by preventing GCP subunit addition beyond position 14 (Wieczorek et al., 2020a, 2020b). It also may regulate nucleation by associating with α/β-tubulin, acting as a “latch” that must be removed for complete γ-TuRC ring closure (Brito et al., 2024). Although previously assigned as MZT1:GCP3-NHD (Würtz et al., 2022; Wieczorek et al., 2020a; Xu et al., 2024), the latch’s identity is unclear. The γ-TuRC contains five copies of GCP3; 4 GCP3-NHDs are found in the NEDD1 pinwheel, and 1 GCP3-NHD is found in the lumenal bridge (Fig. 1 D). Therefore, the latch density must correspond to another MZT:GCP-NHD module.

Using AlphaFold, we predicted structures of GCP3 with various MZT modules. The GCP3:MZT1:GCP5-NHD co-complex fit well with the seam density in our γ-TuRC and prior reconstructions (Würtz et al., 2022; Wieczorek et al., 2020a; Zimmermann et al., 2020), unlike the GCP3:MZT1:GCP3-NHD prediction (Fig. 3, B and C). The fitted MZT1:GCP5-NHD model explains a chemical cross-link between GCP5 K100 and γ-tubulin K410 in the native γ-TuRC (Consolati et al., 2020) (Fig. S3 D). The ∼80-residue (∼23 nm) flexible linker between GCP5’s GRIP1 domain and its NHD satisfies the ∼14-nm Euclidean distance between these two domains in the γ-TuRC (Fig. 3, D and E). Our NEDD1-bound γ-TuRC structure identifies the latch as MZT1:GCP5-NHD, allowing GCP5 to contribute to multiple long-range γ-TuRC interfaces, similar to GCP6’s role in both the lumenal bridge and belt in stabilizing GCP2/3 subunits across the ∼30-nm–wide complex.

### The γ-TuRC can accommodate both a CDK5RAP2-containing CMG module and the NEDD1 pinwheel

Muroyama et al. (2016) proposed two populations of γ-TuRCs in cells: NEDD1-associated, functioning in microtubule anchoring, and CDK5RAP2-associated, promoting microtubule nucleation (Muroyama et al., 2016). However, both NEDD1 and CDK5RAP2 function in γ-TuRC centrosomal localization (Choi et al., 2010; Lüders et al., 2006). Notably, the NEDD1 pinwheel does not block known CDK5RAP2-binding sites between GCP2 GRIP1 and GRIP2 domains, including at position 13 (Fig. 1 D) (Wieczorek et al., 2020a, 2020b; Xu et al., 2024; Serna et al., 2024). Thus, NEDD1 and CDK5RAP2 can likely bind the γ-TuRC simultaneously, as hypothesized (Muroyama et al., 2016), though their combined impact on the γ-TuRC’s conformational variability remain unclear (Xu et al., 2024; Serna et al., 2024).

**Figure 4.**
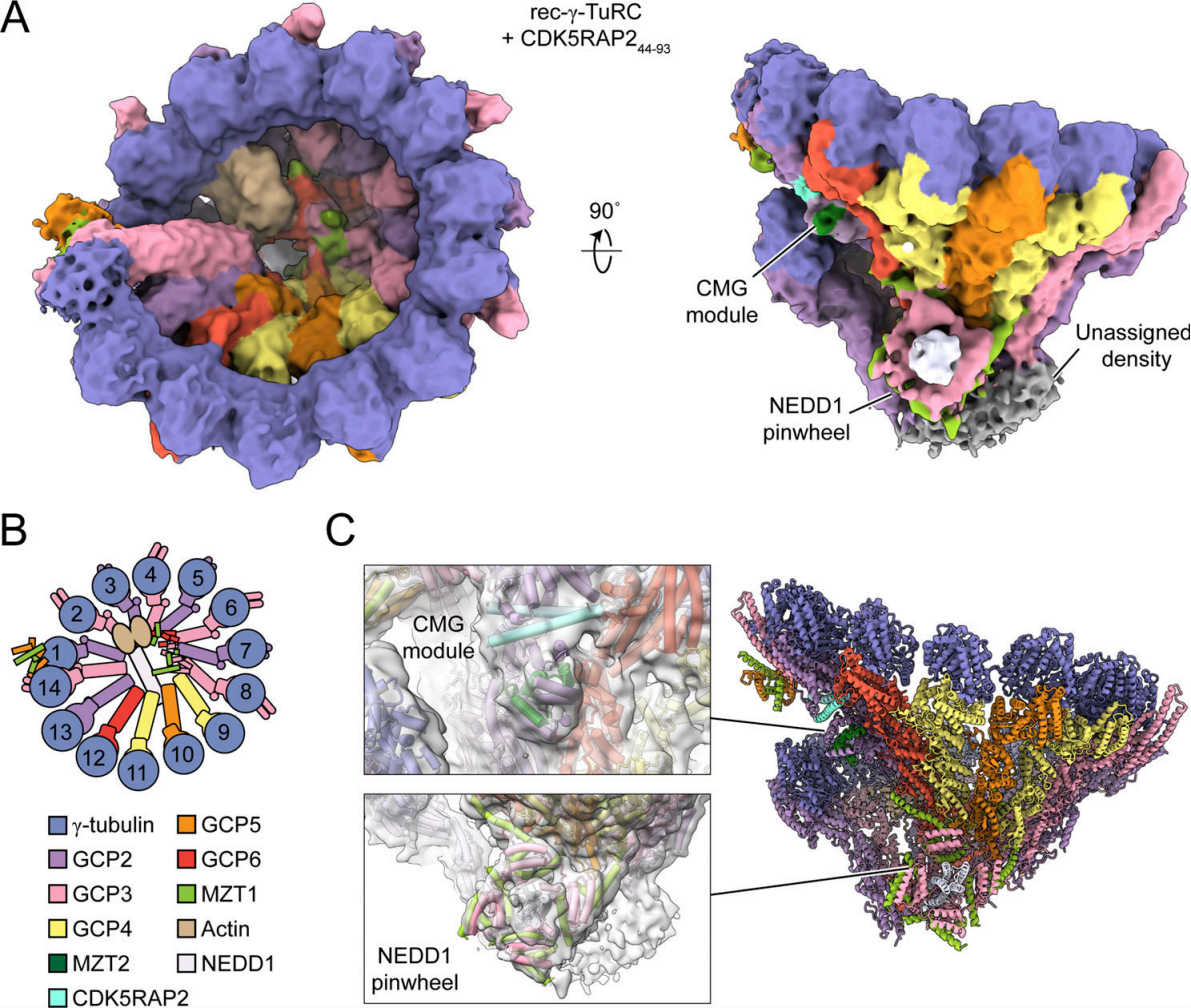
**A fragment of CDK5RAP2 can associate with the NEDD1-bound γ-TuRC. (A)** Two views of the consensus rec-γ-TuRC + CDK5RAP2 density map (surface representation). Unassigned, NEDD1 pinwheel and CMG module densities are indicated. Map resolution is 5.1 Å but is shown at a low threshold to include features with weaker density. **(B)** A schematic top view of the γ-TuRC’s subunit organization. **(C)** Side view of a refined molecular model of the rec-γ-TuRC + CDK5RAP2 (cartoon representation), with zoomed in views for the CMG module at position 13 and NEDD1 pinwheel in the density (transparent surface).

To address this, we collected a large cryo-EM dataset of rec-γ-TuRC with CDK5RAP2 residues 44–93, encompassing the so-called γ-TuRC nucleation–activating motif (Table S1) (Choi et al., 2010; Xu et al., 2024). Using a similar processing pipeline as for rec-γ-TuRC, we increased the CDK5RAP2-bound rec-γ-TuRC reconstruction resolution from 11 to 5.1 Å (Fig. S2 C) (Xu et al., 2024). Gratifyingly, we also observed a pinwheel density in the new 3D reconstructions. Focused 3D classification yielded a clearer NEDD1 pinwheel density map at 6.9-Å resolution (Fig. 4 A; Fig. S2, C, D, G, and H; and Table S2). Prior work showed that CDK5RAP2 induces CDK5RAP2:MZT2:GCP2-NHD (CMG) module formation only at rec-γ-TuRC position 13 (Xu et al., 2024). Our reconstructions confirmed this CMG module persists in the presence of the NEDD1 pinwheel density (Fig. 4, A and B). We built a molecular model of rec-γ-TuRC decorated by both CDK5RAP2 and NEDD1 (Fig. 4 C and Table S3), showing that the NEDD1 pinwheel retains its structure without disrupting CMG module formation (Fig. 4 C).

### NEDD1 does not significantly alter the conformation of the γ-TuRC

**Figure 5.**
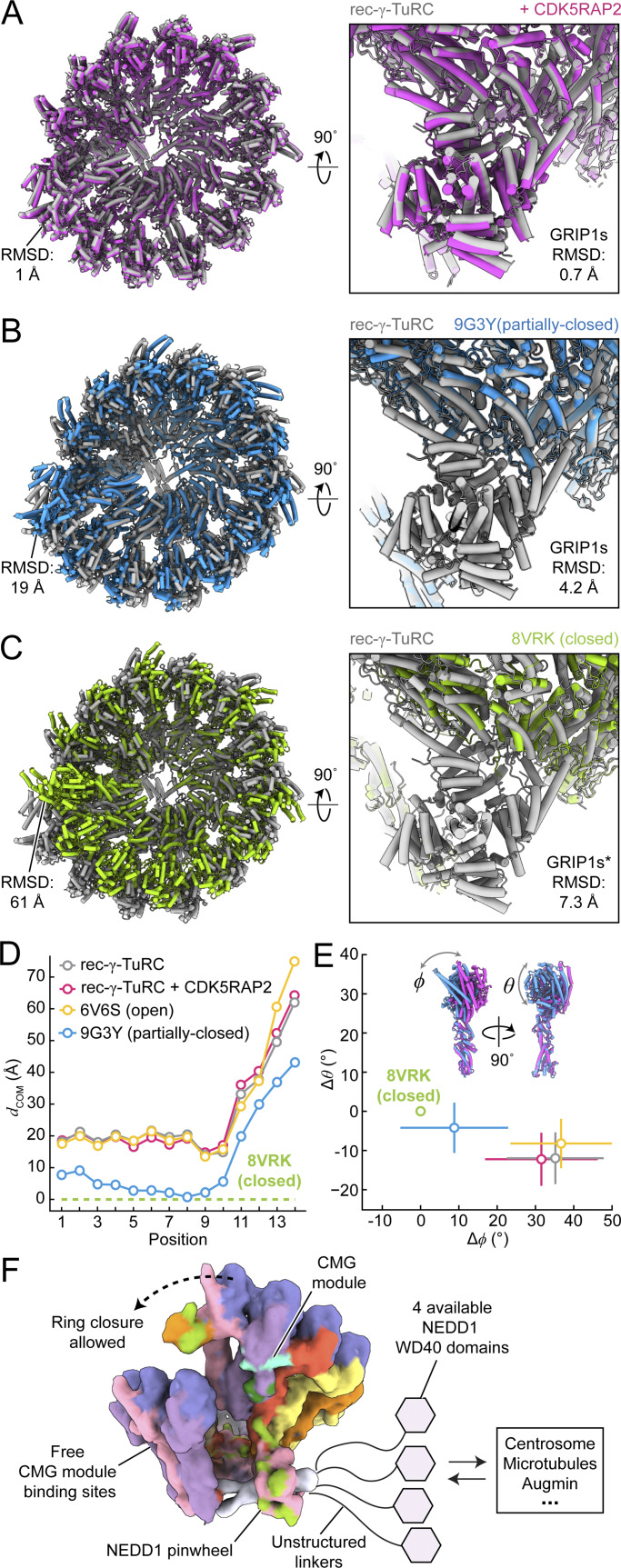
**NEDD1 does not influence the conformation of the γ-TuRC. (A)** Two cartoon representation views of superimposed rec-γ-TuRC (grey) and rec-γ-TuRC + CDK5RAP2 (magenta) models determined in this study. The right panel shows a close-up view of the NEDD1 pinwheel and its binding interface with the GRIP1 domains of GCP4–6 at positions 9–12. **(B)** Two cartoon representation views of superimposed rec-γ-TuRC (grey) and a “partially-closed”, CMG-decorated γ-TuRC (blue; PDB: 9G3Y [Xu et al., 2024]). The right panel shows a close-up view of the NEDD1 pinwheel and its location relative to the GRIP1 domains of GCPs modeled at positions 9–12. **(C)** Two cartoon representation views of superimposed rec-γ-TuRC (grey) and the “fully-closed” rec-γ-TuRC (green; PDB: 8VRK [Aher et al., 2024]). The right panel shows a close-up view of the NEDD1 pinwheel and its location relative to the GRIP1 domains of GCPs modeled at positions 9–12. RMSD values for γ-tubulins at position 14 (left) and position 9–12 GCP GRIP1 domains (right) are indicated in panels A–C. Asterisk in panel C is to clarify that the GRIP1 domains in 8VRK correspond not to GCP4/5/6 but to GCP2/3 models, built into an 8.5-Å reconstruction (Aher et al., 2024), both of which might potentially limit the accuracy of the RMSD measurement in this example. **(D)** Plot of Euclidean center of mass distances (dCOM) versus γ-TuRC subunit position between the indicated γ-TuRC models (rec-γ-TuRC, rec-γ-TuRC + CDK5RAP2, PDB: 6V6S as the open conformation (Wieczorek et al., 2020b), and the “partially-closed,” CMG-decorated (Xu et al., 2024), all relative to PDB: 8VRK, corresponding to a model of the closed rec-γ-TuRC (Aher et al., 2024). **(E)** Plot of the average shift in θ versus the shift in ϕ for helix H12 in γ-tubulins from each γ-TuRC described in D, relative to γ-tubulins at the same positions in the closed γ-TuRC (green circle, indicated). Standard errors in ϕ and θ are displayed as lines. The axes in E are scaled equally. Coloring in E follows the legend in D. **(F)** Model summarizing the findings in this study. The rec-γ-TuRC + CDK5RAP2 model has been converted to a 15-Å low-pass filtered map and colored according to Fig. 4 B. Unresolved WD40 domains stemming from the NEDD1 pinwheel and available to interact with centrosomes, microtubules, augmin, and/or other partners are shown as hexagons. Free CMG module–binding sites that should still be able to induce γ-tubulin ring closure in the presence of the NEDD1 pinwheel are indicated. RSMD, root mean squared deviation.

We next investigated the impact of NEDD1 binding on γ-TuRC conformation. Comparing rec-γ-TuRC models with and without CDK5RAP2 showed no significant differences in subunit organization between the two NEDD1-bound complexes (Fig. 5 A). Notably, both models adopt the open γ-TuRC conformation, based on γ-tubulin:γ-tubulin distances and GRIP2 domain rotation angles (Fig. 5, D and E), relative to the “closed” rec-γ-TuRC conformation derived from microtubule end-capped reconstructions (Aher et al., 2024). Superimposing NEDD1-bound rec-γ-TuRC with the closed rec-γ-TuRC (Aher et al., 2024) or the partially closed, CMG-decorated γ-TuRC (Xu et al., 2024) yielded root mean squared deviation values of <7.5 Å for GCP GRIP1 domains at positions 9–12 (Fig. 5, B and C), which is comparable to our cryo-EM resolution limits, indicating no major clashes at the NEDD1 pinwheel interfaces during γ-TuRC conformational activation. Thus, CDK5RAP2 and NEDD1 can simultaneously bind to the γ-TuRC, and NEDD1 is not predicted to significantly affect γ-TuRC conformational changes during ring closure.

We have determined the cryo-EM structure of NEDD1 bound to the γ-TuRC, revealing that NEDD1’s C terminus forms a tetrameric helical bundle that interfaces with the GRIP1 domains of GCP2 and GCP3 near the γ-TuRC seam. Four MZT1:GCP3-NHD subcomplexes encircle the NEDD1 tetramer to form a pinwheel structure that docks onto GCP4, GCP5, and GCP6, with extensive contacts mediated in part by two distinct α-helical elements in GCP5 and GCP6 (Fig. 2, H and I; and Fig. S3 E). We have assigned several densities in the γ-TuRC, including (1) an extension of the GCP6 belt contacting NEDD1’s fishtail, (2) a GCP5 GRIP1 insertion binding the lumenal face of GCP6, and (3) a MZT1:GCP5-NHD module at the γ-TuRC seam.

Despite using full-length NEDD1, only its C-terminal α-helices are resolved in our reconstructions. The pinwheel structure positions NEDD1’s N-terminal WD40 repeat domains away from the γ-TuRC and allows them to interact with microtubules and augmin for branching nucleation (Fig. 5 F) (Uehara et al., 2009; Zhang et al., 2022). Four NEDD1 molecules per γ-TuRC may also explain oligomeric clustering of augmin at reconstituted branch sites (Zhang et al., 2022). With the exception of S637 at the fishtail, NEDD1 phosphorylation sites (S377, S405, S411, and the region of S557–S574) all lie in the unresolved tether that may form an additional augmin-binding region (Lüders et al., 2006; Pinyol et al., 2013; Sdelci et al., 2012; Gomez-Ferreria et al., 2012; Zhang et al., 2022). The γ-TuRC seam region may thus serve as a microtubule/augmin-binding site.

The NEDD1 pinwheel does not influence the γ-TuRC’s open conformation. Instead, its large buried surface area supports NEDD1’s role as a mechanical anchor, potentially orienting nucleated microtubules (Muroyama et al., 2016). The pinwheel also allows CMG module formation on the γ-TuRC—at least at position 13, explaining how NEDD1 and CDK5RAP2, key γ-TuRC attachment factors, coordinate γ-TuRC localization and activation at microtubule-organizing centers (Muroyama et al., 2016; Fong et al., 2008). Although CMG modules were not well-resolved at γ-TuRC positions 3, 5, or 7, as recently observed for other γ-TuRCs (Xu et al., 2024; Serna et al., 2024), the NEDD1 pinwheel binds only GCP4/5/6 GRIP1 domains on the opposite side of the complex. Conformational analysis (Fig. 5, A–E) showed no clashes between the NEDD1 pinwheel and GRIP1 domains in the closed rec-γ-TuRC structure, suggesting that NEDD1 permits the rotation of γ-tubulin and GCP GRIP2 domains toward partially (Xu et al., 2024; Serna et al., 2024) and fully closed γ-TuRC configurations (Brito et al., 2024; Aher et al., 2024) (Fig. 5 F), consistent with recent in situ studies of NEDD1- and CMG-decorated γ-TuRCs in centrosomes (Gao et al., 2025).

## Materials and methods

### Purification of rec-human γ-TuRC

Polycistronic donor plasmid coding for human γ-tubulin, GCP2 and GCP3 (pACEBac1-γ-TuSC; plasmid # 178079; Addgene; https://n2t.net/addgene:178079; RRID:Addgene_178079) and human γ-tubulin, GCP2, GCP3, GCP4, GCP5, GCP6, MZT1, ZZ-TEV-MZT2-3C-mEGFP, and actin (pACEBac1-γ-TuRC-GFP; plasmid # 178074; Addgene; https://n2t.net/addgene:178074; RRID:Addgene_178074) were transformed into DH10MultiBacTurbo cells (ATG:biosynthetics GmbH), and transposition-positive colonies were selected and used to generate recombinant bacmids. Bacmids were transfected into Sf9 cells (Novagen) following the Bac-to-Bac manual (Invitrogen), baculoviruses were amplified twice, and fresh virus from γ-TuRC-GFP and γ-TuSC bacmids were mixed together at a 1:1 ratio. This virus mixture was used to infect 2 liters of High Five cells (Thermo Fisher Scientific) at a cell density of 3 × 10^6^ per ml for 60 h at 27°C. Cells were harvested by centrifugation at 1,000 *g*, resuspended in 60 ml ice-cold lysis buffer (40 mM HEPES, pH 7.5, 150 mM KCl, 1 mM MgCl_2_, 10% glycerol [vol/vol], 0.1% Tween-20, 0.1 mM ATP, 0.1 mM GTP, 1 mM 2-mercaptoethanol, one cOmplete EDTA-free Protease Inhibitor Cocktail tablet [Roche], and 2 mM PMSF) and lysed by dounce homogenization on ice. The lysate was clarified at 322,000 *g* for 1 h at 4°C, 0.22-µm syringe filtered, and loaded onto a 1-ml NHStrap column (Cytiva) previously coupled to 10 mg rabbit IgG (IRBIGGAP500MG; Innovative Biosciences) following the manufacturer’s instructions. The IgG column was washed with lysis buffer followed by gel filtration buffer (40 mM HEPES, pH 7.5, 150 mM KCl, 1 mM MgCl_2_, 10% glycerol [vol/vol], 0.1 mM GTP, and 1 mM 2-mercaptoethanol). An expression vector for TEV protease, pRK793, was a gift from David Waugh (plasmid 8827; Addgene; https://n2t.net/addgene:8827; Research Resource Identifier: Addgene_8827; [Kapust, 2001]). TEV was expressed in BL21-CodonPlus (DE3)-RIL and purified using Ni-NTA and gel filtration following the methods described in Ti et al. (2020). 1 mg of TEV protease (stored in 40 mM HEPES, pH 7.5, 30% [wt/vol] glycerol, 150 mM KCl, 1 mM MgCl_2_, and 3 mM 2-mercaptoethanol) was diluted into 1 ml of gel filtration buffer and injected onto the IgG column, and proteolysis was allowed to proceed for 2 h at 4°C. The digested eluate was pooled, concentrated with a 100-kDa cutoff spin filter (Millipore), and gel filtered over a Superose 6 Increase 10/300-GL column (Cytiva) pre-equilibrated in gel filtration buffer. Peak fractions were pooled and loaded onto two 2-ml sucrose gradients composed of 10%, 20%, 30%, and 40% sucrose (wt/vol) in gradient buffer (40 mM HEPES, pH 7.5, 150 mM KCl, 1 mM MgCl_2_, 0.01% Tween-20 [vol/vol], 0.1 mM GTP, and 1 mM 2-mercaptoethanol). The gradient was centrifuged at 50,000 rpm in a TLS-55 rotor at 4°C for 3 h with minimum acceleration and no break. Fractions were manually collected with a cut-off P1000 pipette tip and analyzed by SDS-PAGE followed by Coomassie staining and/or negative-stain TEM. Peak fractions were aliquoted, snap-frozen, and stored in liquid N2. Gradients were fractionated into 250 μl and analyzed by SDS-PAGE followed by Coomassie staining (Xu et al., 2024).

### Purification of recombinant, untagged CDK5RAP2 fragments

A bacterial expression construct for aa 44–93 of CDK5RAP2 lacking a GFP tag was previously described (Xu et al., 2024). The plasmid was transformed into BL21(DE3) pRIL *Escherichia coli* cells (Stratagene), and His6-SUMO-TEV-CDK5RAP2_44–93_ expression was induced with 0.5 mM IPTG for 16 h at 18°C. Cell pellets from 3 L culture were resuspended in 45 ml Ni-NTA lysis buffer (50 mM sodium phosphate, pH 8.0, 300 mM NaCl, 15 mM imidazole, 0.1% [vol/vol] Tween-20, and 1 mM 2-mercaptoethanol) and lysed by three passes through an Emulsiflex C-5 (Avestin). Lysate was clarified at 35,000 rpm in a Type 45 Ti rotor (Beckman) for 45 min at 4°C, and the supernatant was mixed with 3 ml Ni-NTA agarose (Qiagen) pre-equilibrated in lysis buffer. The resin was then washed extensively with lysis buffer. Protein was eluted with Ni-NTA elution buffer (lysis buffer containing an additional 485 mM imidazole). Peak fractions were identified by Bradford assay, pooled, concentrated to ∼2.5 ml with a 3-kDa cutoff spin filter (Millipore), and applied to PD-10 desalting column (Cytiva) pre-equilibrated with lysis buffer to remove imidazole. The elution was incubated with 1 mg TEV protease for 2 h at 4°C, and injected into a 1-ml HisTrap column (Cytiva) pre-equilibrated with lysis buffer. Peak fractions were identified by Bradford assay, pooled, concentrated to ∼1 ml at ∼50 mg/ml with the 3-kDa cutoff spin filter (Millipore). Untagged CDK5RAP2_44–93_ was then further purified over a HiLoad 16/600 Superdex 75 column pre-equilibrated in gel filtration buffer (40 mM HEPES, pH 7.5, 150 mM NaCl, 1 mM MgCl_2,_ and 2 mM 2-mercaptoethanol). Peak fractions were identified by SDS-PAGE, pooled, concentrated, supplemented with sucrose to 10% (wt/vol), aliquoted, snap frozen in liquid nitrogen, and stored at −80°C. After TEV cleavage, the resulting CDK5RAP2_44–93_ peptide contains an N-terminal SNIGSGGTPSTGSSLPNVSEE sequence as a remnant of the linking sequence between the TEV cleavage site and the multiple cloning site in the modified expression vector. LC-MS/MS was used to confirm the identity of purified CDK5RAP2_44–93_, as well as a lack of phosphorylated peptides, as expected for a bacterially expressed protein (Xu et al., 2024).

### Cryo-EM sample preparation

EM grids were prepared with Ultrathin Carbon Film on a Lacey Carbon Support Film (400 mesh; Copper Ted Pella, Inc) or by overlaying Quantifoil 300 mesh (copper, R 2/2) holy carbon grids with homemade continuous carbon film. Lacey grids were glow discharged at 30 mA for 45 s and at 15 mA for 15 s for Quantifoil grids prepared with homemade carbon support films. Following, the grids were transferred using metal forceps onto an ice-cold block. rec-γ-TuRC samples were preincubated with 2 μM CDK5RAP2_44–93_ for 10 min on ice prior to grid application. A 2.5 μl aliquot of mixed sample was applied to the grid for 5 min, followed by manual blotting. This process was repeated eight times. After the final application, the grid was washed by incubating twice with 20 μl of washing buffer (40 mM HEPES, pH 7.5, 150 mM KCl, 1 mM MgCl_2_, 1 mM 2-mercaptoethanol, 0.01% Tween-20, and 0.1 mM GTP) for 1 min. An additional 3.5 μl of washing buffer was applied onto the grid. The grid was transferred to a Vitrobot Mark IV (Thermo Fisher Scientific), blotted for 4 s with a blot force of −1 at 100% humidity and 4°C, plunge frozen in a liquid ethane/propane mixture, and stored in liquid nitrogen until screening in the ScopeM facility at ETH (Zürich, Switzerland).

### Cryo-EM data collection

For rec-γ-TuRC + CDK5RAP2, 192,360 new movies were collected on a TFS Titan Krios G3i (2) FEG operated with a Gatan K3 in CDS mode and a slit width of 20 eV on a GIF BioQuantum energy filter. These datasets were combined with four previously described datasets for analysis (Xu et al., 2024). Full cryo-EM data collection statistics are listed in Table S2. Automatic data collection was performed with “Faster acquisition mode” in EPU software (Thermo Fisher Scientific).

### Cryo-EM image processing

#### rec-γ-TuRC

Particles were first autopicked in RELION 4.0 from micrographs in Aher et al. (2024) using 2D classes generated from a small subset of manually picked γ-TuRC particles as picking templates. Autopicked particles were then subjected to multiple rounds of 2D and 3D classification to form a roughly cleaned stack of 1,990,604 γ-TuRC particles. The particles were imported into CryoSPARC and classified by heterogeneous refinement using two copies of γ-TuRC averaged from the particle stack and five “noise” density maps generated by premature cancellation of a 90-particle ab initio refinement as references. This was performed in multiple rounds until minimal proportions of particles were being assigned to the noise classes, revealing a subset of 584,330 particles. A final heterogeneous refinement was performed using three copies of an average of the particle subset, as well as three copies of the average of the whole dataset, revealing a subset of 266,675 particles with better quality γ-TuRC density. The final particles were subjected to local refinement using a γ-TuRC–shaped mask to generate the final consensus rec-γ-TuRC reconstruction at 4.7-Å resolution (Fig. S2, A, B, E, and F).

We also tested different software tools to analyze the heterogeneity and occupancy of the NEDD1 pinwheel, including those developed in RELION-5 (Blush regularization, DynaMight, and MultiBody refinements [Burt et al., 2024, *Preprint*]), cryoDRGN (Zhong et al., 2021), and OccuPy (Forsberg et al., 2023), none of which significantly improved tertiary or secondary structure features within the pinwheel density.

#### rec-γ-TuRC + CDK5RAP2

6,000 movies from each dataset were first used to estimate a gain reference for each rec-γ-TuRC + CDK5RAP2 dataset using “relion_estimate_gain” (Burt et al., 2024, *Preprint*). Movies were then imported into CryoSPARC for patch motion correction and patch contrast transfer function estimation (Punjani et al., 2017). A blob picker with an elliptical blob size of 280–380 Å was used to obtain a curated set of particles, selected through 2D classification and visual inspection of micrographs. Exposures were chosen based on contrast transfer function fit resolution (2–18 Å), astigmatism (0–2,000 Å), and relative ice thickness (0–1.8).

A ResNet16 neural network was trained on the curated particle set extracted from 16× downsampled micrographs with TOPAZ (Bepler et al., 2020). TOPAZ models were generated for each independent dataset, and the software was used to pick and extract particles using a box size of 196. The TOPAZ-picked particles were subjected to 2D classification in CryoSPARC, and particles were selected based on 2D classes displaying the distinctive γ-TuRC shape.

To increase particle numbers, TOPAZ training and picking were repeated using the selected particles. Selected particles were extracted using a box size of 848 pixels, binned to 144 or 256 pixels, and subjected to 2D classification. Any repeating particle coordinates within 200 Å of each other were removed using the “remove duplicates” feature in CryoSPARC. The resulting 296,981 particles were then subjected to ab initio reference generation expecting three classes. However, only one γ-TuRC–like class resulted from this run; the other two classes converged as noise references. All particles were then subjected to heterogeneous refinement with “force hard classification” turned on and using the ab initio maps as starting references. The particles classified into the γ-TuRC–like class were re-extracted at a box size of 384 pixels and subjected to nonuniform refinement.

To generate a consensus reconstruction of rec-γ-TuRC + CDK5RAP2 with improved NEDD1 density, 3D classification was performed in CryoSPARC, with a mask focusing on the pinwheel-shaped density. This classification, using two classes and filtered at 12 Å, aimed to uncover rare or hidden conformations. The first class contained 77,282 particles, while the second had 71,778 particles. Both classes were independently refined via local refinement, producing a final consensus map of the rec-γ-TuRC + CDK5RAP2 complex with well-resolved NEDD1 pinwheel density from the second class (Fig. S2, C, D, G, and H).

### Model building

For the rec-γ-TuRC model, a combination of structure predictions were first generated using AlphaFold 3 (Abramson et al., 2024). For instance, NEDD1 (UniProt accession: Q8NHV4), MZT1 (UniProt accession: Q08AG7), and GCP3-NHD (UniProt accession: Q96CW5) protein sequences were used to assemble the pinwheel (Fig. 1 C). GCP2 (UniProt accession: Q9BSJ2), GCP3 (UniProt accession: Q96CW5), GCP4 (UniProt accession: Q9UGJ1), GCP5 (UniProt accession: Q96RT8), GCP6 (UniProt accession: B2RWN4), and MZT1 (UniProt accession: Q08AG7), together with γ-tubulin (UniProt accession: P23258):GCP3:MZT1:GCP5-NHD and γ-tubulin:GCP3:MZT1:GCP3-NHD, were all used to interpret the consensus map. Lumenal bridge components were similarly predicted with AlphaFold 3.

The atomic model of the native human γ-TuRC (PDB ID: 6V6S) was fitted into the rec-γ-TuRC consensus map (Fig. 1 D). The different AlphaFold predictions were initially aligned to this model using the “matchmaker” tool in ChimeraX and then manually corrected in Coot. Due to insufficient resolution for model building, side chains were trimmed to β-carbons, and the resulting model was real-space refined in PHENIX (Afonine et al., 2018).

For the rec-γ-TuRC + CDK5RAP2 model, a starting AlphaFold 3 structure containing GCP2, GCP6, MZT2 (Q6NZ67), and two copies of residues 44–93 of CDK5RAP2 (I3LKY1) was docked into the rec-γ-TuRC + CDK5RAP2 map using the “fit in map” function of UCSF ChimeraX (Goddard et al., 2018). All other subunits originated from the rec-γ-TuRC model above. Model domains were positioned into corresponding local density using the “Fit all chains to Map” tool in Coot, and any loops or structural features not supported by local density were removed. Due to insufficient resolution for model building, side chains were trimmed to β-carbons, and the resulting model was real-space refined in PHENIX (Afonine et al., 2018).

Refinement statistics for rec-γ-TuRC and rec-γ-TuRC + CDK5RAP2 models are available in Table S3. Only RELION or CryoSPARC auto-sharpened EM density maps were used for final model refinements in PHENIX.

### Pull-down experiments

Mammalian expression pcDNA3.1 vectors encoding WT, E598A/D602A/E605A, F603A/F622A, and 1–634 NEDD1 with N-terminal Myc-SBP (streptavidin-binding peptide) tag, as well as the GCP6 WT and 329–341 deletion plus the GCP5 WT and quadruple mutant R213A/R228G/L256E/V258E with C-terminal SBP-Myc tag, were synthetized via gene synthesis (Genewiz). The canonical NEDD1, GCP6, and GCP5 protein isoforms with UniProt accession numbers Q8NHV4-1, Q96RT7–1, and Q96RT8–1 were used, respectively. N-terminally V5-tagged WT NEDD1 in pcDNA3.1 was cloned via the Q5 Site-Directed Mutagenesis Kit (New England Biolabs) using the following oligos: V5-NEDD1 fwd = 5′ CTG​CTG​GGC​CTG​GAT​AGC​ACC​AGA​AGC​AGA​GGC​ATG-3′; V5-NEDD1 rev = 5′-CGG​GTT​CGG​AAT​CGG​TTT​GCC​CAT​GAA​TTC​CAC​CAC​AC-3′. 2.5–3 × 10^6^ HEK293T cells, cultured in DMEM containing 10% FBS and 1X penicillin/streptomycin, were seeded in 10-cm plates and transfected 24 h later using PEI Max (1:3 of DNA-to-PEI mass ratio). Cells were transfected with 20 µg of total plasmid, with a 1:3 V5-NEDD1 to GCP-SBP-Myc DNA ratio in co-transfection experiments. 24 h later, cells were harvested and lysed for 30 min at 4°C with lysis buffer while rotating. For detection of endogenous γ-tubulin in NEDD1 pulldowns, lysis buffer containing 50 mM Tris-HCl, pH 7.5, 150 mM NaCl, 0.1% Triton X-100, 10% glycerol, and 2 mM DTT was used, while for detection of co-transfected V5-NEDD1 in GCP pulldowns, lysis buffer containing 50 mM HEPES, pH 7.5, 150 mM NaCl, 1% NP-40, 0.5% sodium deoxycholate, and 2 mM DTT was used. All buffers were supplemented with EDTA-free protease inhibitor (Roche), 1 mM sodium fluoride, 1 mM *β*-glycerophosphate, and 1 mM sodium pyrophosphate. Crude lysates were centrifuged at 16,000 *g* for 15 min at 4°C and their total protein amount was normalized with the Bradford protein assay. The cleared lysate was rotated for 1 h at 4°C in presence of 50 μl of Streptavidin Sepharose High Performance (Cytiva). Beads were washed three times with lysis buffer. Bound proteins were eluted with 50 μl 2x SDS protein sample buffer. For further analysis, proteins were separated by SDS-PAGE and detected by western blotting. c-Myc monoclonal antibody (clone 9E10, Cat# MA1–980; Invitrogen), anti–γ-tubulin monoclonal antibody (clone GTU-88, Cat# T5326; Sigma-Aldrich), and V5 Tag monoclonal antibody (clone SV5-Pk1, Cat# R960-25; Invitrogen) were used at 1:2,000, 1:1,000 and 1:5,000 dilutions, respectively. HRP-conjugated goat anti-mouse IgG secondary antibody (Cat#31430; Invitrogen) was used at 1:5,000. Immun-Blot PVDF Membrane (Cat #1620177; Bio-Rad) was used for all western blots. Untransfected cells and V5-NEDD1 transfected cells served as negative control in NEDD1 and GCP pulldowns, respectively. Chemiluminescent images were acquired using a Bio-Rad ChemiDoc system; blots in figures display scaling from “raw” image outputs, while uncropped blots in Source Data display both raw and .tif output files (to show 300-dpi resolutions).

### Helical parameter analysis

Models for rec-γ-TuRC, rec-γ-TuRC + CDK5RAP2, and the human native γ-TuRC in the open conformation (PDB 6V6S; [Wieczorek et al., 2020b]) were aligned to a structure of γ-TuRC bound to the microtubule end (PDB 8RVK; [Aher et al., 2024]) via the GRIP1 domains of GCP3 at positions 2, 4, 6, and 8 using the align command in PyMoL. The aligned structures were subsequently read out into PDB format and analyzed using a set of previously described custom python scripts (Xu et al., 2024). Specifically, the aligned PDB files were loaded, and coordinates of all Cα atoms in the structures were extracted using biophython (Cock et al., 2009). The position of each γ-tubulin was given by the center of mass (CoM), i.e., the mean of its Cα atoms. The CoM was used to analyze structural changes due to its robustness and stability concerning conformational changes of individual γ-tubulins. The Euclidean distance between the CoM of the reference rec-γ-TuRC in the closed conformation and all other γ-TuRC structures at each position was calculated to analyze changes between complexes.

To examine changes in the orientation of individual γ-tubulins across the structures, the CoM of the Cα′s of the first half and second half of γ-tubulin helix 12 was used to define a vector describing tilt and rotation of each γ-tubulin. The coordinates were transformed to spherical coordinates, where phi (ϕ) denotes the angle of the vectors projected in x-y plane, while theta (Ө) describes the tilt away from the z axis (the length of the vectors are set to 1 for simplicity). Normalization of theta and phi was done by subtraction, resulting in the direction of the change in orientation of each γ-tubulin with respect to the closed rec-γ-TuRC reference structure.

### Online supplemental material

Fig. S1 shows the AlphaFold predictions and NEDD1 conservation analysis. Fig. S2 shows the cryo-EM processing pipeline. Fig. S3 shows the details regarding rec-γ-TuRC consensus reconstruction and model building. Table S1 shows the cryo-EM data collection. Table S2 shows the cryo-EM data processing statistics. Table S3 shows the model building and refinement statistics. Table S4 shows the (interface) predicted template modeling (ipTM and pTM) scores of AlphaFold predictions.

## Supplementary Material

Table S1 shows the cryo-EM data collection.

Table S2 shows the cryo-EM data processing statistics.

Table S3 shows the model building and refinement statistics.

Table S4 shows the (interface) predicted template modeling (ipTM & pTM) scores of AlphaFold predictions.

SourceData F2 is the source file for Fig. 2.

SourceData FS3 is the source file for Fig. S3.

## Data Availability

Models and cryo-EM density maps have been deposited to the PDB (PDB ID: 9QVM and 9QVN) and EMDB (EMDB-53339 and EMDB-53400). Uncropped western blots are included in the Source Data. All other data are available on reasonable request.
