# Correction: Structure of the microtubule-anchoring factor NEDD1 bound to the γ-tubulin ring complex

**DOI:** 10.1083/jcb.20241020606232026c

**Published:** 2026-07-22

**Authors:** Hugo Muñoz-Hernández, Yixin Xu, Aitor Pellicer Camardiel, Daniel Zhang, Allen Xue, Amol Aher, Ellie Walker, Florina Marxer, Tarun M. Kapoor, Michal Wieczorek

Vol. 224, No. 8 | https://doi.org/10.1083/jcb.202410206 | May 21, 2025

After publication, the authors noticed errors in figure citations and figure legends. These have been corrected as shown below; additions are indicated in bold, and deletions are crossed out.

In the Results and discussion section “The NEDD1 pinwheel associates with the γ-TuRC through conserved interfaces,” the citation for Fig. 1 F has been changed to Fig. 1, E and F, in the first sentence of the first paragraph. In the second paragraph, citations for Fig. 2 G have been changed to Fig. 2 H in the second and third sentences, and a citation for Fig. S3 has been deleted.

## The NEDD1 pinwheel associates with the γ-TuRC through conserved interfaces

We next used our density maps and AlphaFold predictions to build a molecular model of the human γ-TuRC bound to the NEDD1 pinwheel (Fig. 1 F;**E and F**; Fig. S3 B and Tables S2 and S3). The refined NEDD1 pinwheel model revealed conserved interfaces with MZT1:GCP3-NHD modules (Fig. S1, I and J). NEDD1 residues F603 and F622 form hydrophobic cores that may be critical for NEDD1 helical assembly (Fig. 2, A and B), and electrostatic interactions between NEDD1 residues E598, D602, and E605 and GCP3-NHD residues K57 and K60 likely stabilize the NEDD1-GCP3-NHD interface (Fig. 2, C and D). Mutating either of these residue sets to alanine (F603A/F622A and E598A/D602A/E605A) both reduced the ability of overexpressed NEDD1 to pull down γ-tubulin from HEK293T cells (Fig. 2 E). These results validate the γ-TuRC-NEDD1 pinwheel model and identify two critical interfaces for NEDD1–γ-TuRC interactions.

We next analyzed the interaction between the NEDD1 pinwheel and the γ-TuRC, which is mediated by two main interfaces. In the first interface, blades A and B of the NEDD1 pinwheel contact the underside of the γ-TuRC (Fig. 2 G**H**). Blade B lies beneath GCP4’s GRIP1 domain at position 9, while blade A interfaces with the GRIP1 domains of GCP5, GCP4, and GCP6 at positions 10–12 (Fig. 2 G**H**). In blade B, MZT1’s C-terminal α-helix (H3) extends along GCP4’s GRIP1 domain (Fig. 2 H). A GCP5 α-helical element (residues ∼243–263) inserts into a pocket in blade B lined by MZT1 α-helices H2 and H3 (Fig. 2, H and I; and Fig. S3 E). In blade A, MZT1 α-helix H3 spans the lower part of GCP4’s and GCP6’s GRIP1 domains, with α-helices formed by GCP5 residues ∼210–220 and GCP6 residues ∼325–343 inserting into a pocket in blade B lined by MZT1 H2 and H3. Mutagenesis of GCP5 hydrophobic residues or partial deletion of the GCP6 α-helix did not reduce NEDD1 levels detected in pulldowns from cultured cells (Fig. S3 E), suggesting redundant binding elements in this large (greater than ∼1,500 Å^2^) interface. These findings clarify how the pinwheel blades bind to the γ-TuRC, with GCP5- and GCP6-specific elements aiding pinwheel orientation, alongside other interfaces (Würtz et al., 2022; Zimmermann et al., 2020).

In “The structure of the NEDD1-bound γ-TuRC enables assignment of GCP5-specific features” section of the Results and discussion, first paragraph, citations for Fig. 3, panels G and H, have been swapped. In the first sentence, the citation for Fig. S3 G has been changed to Fig. S3 H. In the second sentence of this paragraph, the citation for Fig. S3 H has been changed to Fig. S3 G.

## The structure of the NEDD1-bound γ-TuRC enables assignment of GCP5-specific features

We identified an unassigned α-helical element along the luminal face of GCP6’s GRIP1 domain (Fig. S3 G**H**), which was noted in previous γ-TuRC reconstructions (Zimmermann et al., 2020; Xu et al., 2024). AlphaFold predicted that an insertion element in GCP5 corresponding to residues ∼567–608 forms a helix-turn-helix motif that contacts a pocket in GCP6’s GRIP1 domain (Fig. S3 H**G**). The model fits well into previous γ-TuRC reconstructions (Zimmermann et al., 2020) and our own (Fig. S3, I and J). GCP5’s insertion loops back toward GCP5, indicating that the remaining densities crossing toward GCP3 and GCP2 at positions 13–14 must constitute a separate polypeptide chain(s) (Zimmermann et al., 2020), though its identity remains unclear due to resolution limits.

In “The γ-TuRC can accommodate both a CDK5RAP2-containing CMG module and the NEDD1 pinwheel” section of the Results and discussion, second paragraph, the citation for Fig. 4 D has been changed to Fig. 4 C in the last sentence.

## The γ-TuRC can accommodate both a CDK5RAP2-containing CMG module and the NEDD1 pinwheel

To address this, we collected a large cryo-EM dataset of rec-γ-TuRC with CDK5RAP2 residues 44–93, encompassing the so-called γ-TuRC nucleation–activating motif (Table S1) (Choi et al., 2010; Xu et al., 2024). Using a similar processing pipeline as for rec-γ-TuRC, we increased the CDK5RAP2-bound rec-γ-TuRC reconstruction resolution from 11 to 5.1 Å (Fig. S2 C) (Xu et al., 2024). Gratifyingly, we also observed a pinwheel density in the new 3D reconstructions. Focused 3D classification yielded a clearer NEDD1 pinwheel density map at 6.9-Å resolution (Fig. 4 A; Fig. S2, C, D, G, and H; and Table S2). Prior work showed that CDK5RAP2 induces CDK5RAP2:MZT2:GCP2-NHD (CMG) module formation only at rec-γ-TuRC position 13 (Xu et al., 2024). Our reconstructions confirmed this CMG module persists in the presence of the NEDD1 pinwheel density (Fig. 4, A and B). We built a molecular model of rec-γ-TuRC decorated by both CDK5RAP2 and NEDD1 (Fig. 4 C and Table S3), showing that the NEDD1 pinwheel retains its structure without disrupting CMG module formation (Fig. 4 D**C**).

In the “NEDD1 does not significantly alter the conformation of the γ-TuRC” section of the Results and discussion, first paragraph, the citation for Fig. 5, A–C, has been changed to Fig. 5, B and C, in the fourth sentence. In the third paragraph of this section, the citation for Fig. 4 E has been changed to Fig. 5 F in the second sentence.

## NEDD1 does not significantly alter the conformation of the γ-TuRC

We next investigated the impact of NEDD1 binding on γ-TuRC conformation. Comparing rec-γ-TuRC models with and without CDK5RAP2 showed no significant differences in subunit organization between the two NEDD1-bound complexes (Fig. 5 A). Notably, both models adopt the open γ-TuRC conformation, based on γ-tubulin:γ-tubulin distances and GRIP2 domain rotation angles (Fig. 5, D and E), relative to the “closed” rec-γ-TuRC conformation derived from microtubule end-capped reconstructions (Aher et al., 2024). Superimposing NEDD1-bound rec-γ-TuRC with the closed rec-γ-TuRC (Aher et al., 2024) or the partially closed, CMG-decorated γ-TuRC (Xu et al., 2024) yielded root mean squared deviation values of <7.5 A for GCP GRIP1 domains at positions 9–12 (Fig. 5, A–C**B and C**), which is comparable to our cryo-EM resolution limits, indicating no major clashes at the NEDD1 pinwheel interfaces during γ-TuRC conformational activation. Thus, CDK5RAP2 and NEDD1 can simultaneously bind to the γ-TuRC, and NEDD1 is not predicted to significantly affect γ-TuRC conformational changes during ring closure.

Despite using full-length NEDD1, only its C-terminal α-helices are resolved in our reconstructions. The pinwheel structure positions NEDD1’s N-terminal WD40 repeat domains away from the γ-TuRC and allows them to interact with microtubules and augmin for branching nucleation (Fig. 4 E**5 F**) (Uehara et al., 2009; Zhang et al., 2022). With the exception of S637 at the fishtail, NEDD1 phosphorylation sites (S377, S405, S411, and the region of S557–S574) all lie in the unresolved tether that may form an additional augmin-binding region (Lüders et al., 2006; Pinyol et al., 2013; Sdelci et al., 2012; Gomez-Ferreria et al., 2012; Zhang et al., 2022). The γ-TuRC seam region may thus serve as a microtubule/augmin-binding site.

In the Materials and methods section “Model building,” the citation for Fig. 1 D has been changed to Fig. 1 C in the second sentence of the first paragraph. In the first sentence of the second paragraph, the citation for Fig. 1 A has been changed to Fig. 1 D.

## Model building

For the rec-γ-TuRC model, a combination of structure predictions was first generated using AlphaFold 3 (Abramson et al., 2024). For instance, NEDD1 (UniProt accession: Q8NHV4), MZT1 (UniProt accession: Q08AG7), and GCP3-NHD (UniProt accession: Q96CW5) protein sequences were used to assemble the pinwheel (Fig. 1 D**C**). GCP2 (UniProt accession: Q9BSJ2), GCP3 (UniProt accession: Q96CW5), GCP4 (UniProt accession: Q9UGJ1), GCP5 (UniProt accession: Q96RT8), GCP6 (UniProt accession: B2RWN4), and MZT1 (UniProt accession: Q08AG7), together with γ-tubulin (UniProt accession: P23258):GCP3:MZT1:GCP5-NHD and γ-tubulin:GCP3:MZT1:GCP3-NHD, were all used to interpret the consensus map. Luminal bridge components were similarly predicted with AlphaFold 3.

The atomic model of the native human γ-TuRC (PDB ID: 6V6S) was fitted into the rec-γ-TuRC consensus map (Fig. 1 A**D**). The different AlphaFold predictions were initially aligned to this model using the “matchmaker” tool in ChimeraX and then manually corrected in Coot. Due to insufficient resolution for model building, side chains were trimmed to β-carbons, and the resulting model was real-space refined in PHENIX (Afonine et al., 2018).

In the legend for Fig. S2 C, “sDK5RAP2” has been changed to “CDK5RAP2.” The figure itself remains unchanged.

**Figure S2.**
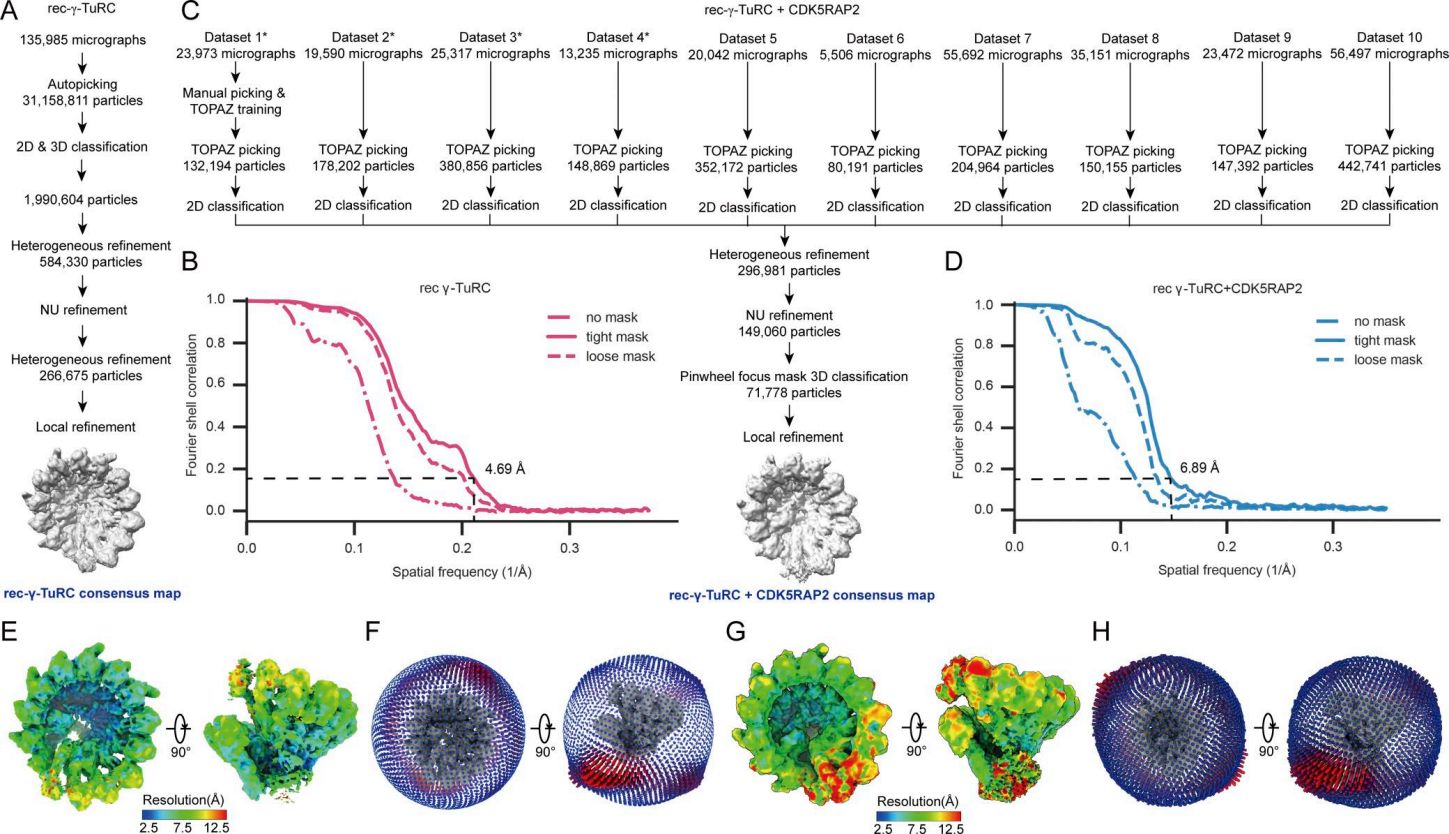
**Cryo-EM processing pipeline. (A)** Summary of the new rec-γ-TuRC cryo-EM data processing strategy. Cryo-EM collection details were reported in Aher et al. (2024). **(B)** Gold-standard Fourier shell correlation (FSC) plot for the consensus rec-γ-TuRC density map. The FSC at 0.143 is indicated by a dashed line. **(C)** Summary of new rec-γ-TuRC + sDK5RAP2**CDK5RAP2** cryo-EM data processing strategy. Cryo-EM collection details for datasets 1–4 (marked by asterisks) were reported in Xu et al. (2024). **(D)** Gold-standard FSC plot for the consensus rec-γ-TuRC + CDK5RAP2 density map. The FSC at 0.143 is indicated by a dashed line. **(E)** Two views of the consensus rec-γ-TuRC density map analyzed by CryoSPARC, showing a resolution distribution ranging from 2.5 to >12.5 Å. **(F)** Two views of the particle angular distribution overlaid onto the rec-γ-TuRC consensus map. **(G)** Two views of the consensus rec-γ-TuRC + CDK5RAP2 density map analyzed by CryoSPARC, showing a resolution distribution ranging from 2.5 to >12.5 Å. **(H)** Two views of the particle angular distribution overlaid onto the rec-γ-TuRC + CDK5RAP2 consensus map.

In the legend for Fig. S3, a portion of the panel G description has been removed. The figure itself remains unchanged.

**Figure S3.**
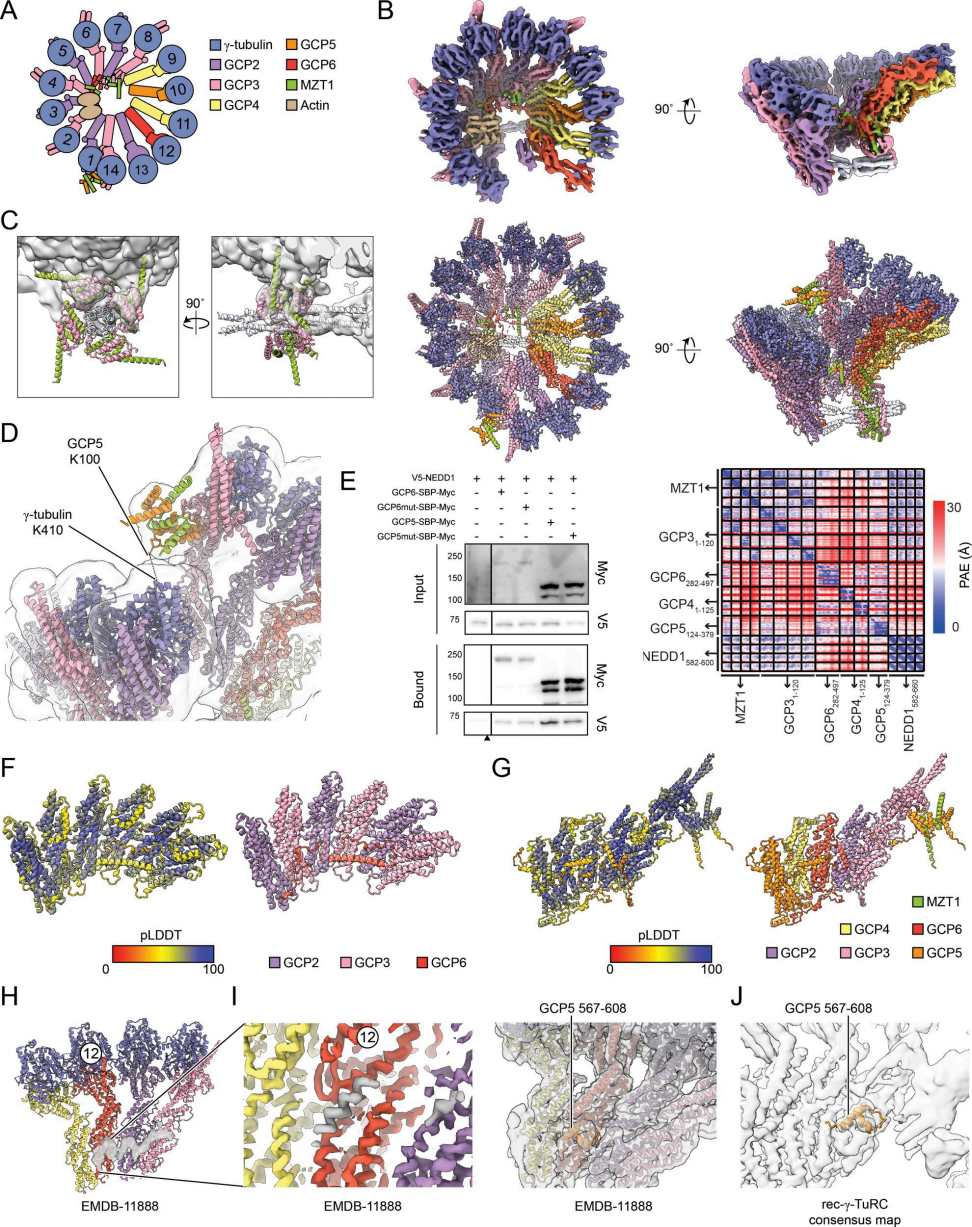
**Details regarding rec-γ-TuRC consensus reconstruction and model building. (A)** Schematic of the γ-TuRC highlighting subunit composition and numbering across the complex. **(B)** Top: two views of the rec-γ-TuRC consensus map showing higher resolution features. The map was sharpened in CryoSPARC and postprocessed with EMready (He et al., 2023). Bottom: two views of the refined rec-γ-TuRC model, including the NEDD1 pinwheel. **(C)** Two views of the NEDD1 pinwheel predicted by AlphaFold 3 (cartoon representation) fitted into the pinwheel density in the rec-γ-TuRC consensus map (transparent surface). **(D)** Cartoon representation view of MZT1:GCP5-NHD at the rec-γ-TuRC seam with the consensus density map in transparent surface representation. GCP5 K100 and γ-tubulin K410, identified as cross-linked residues in the native human γ-TuRC, are indicated (Consolati et al., 2020). **(E)** Left: western blot of inputs and bound fractions of SBP pulldowns of GCP-SBP-Myc constructs from HEK293T cells. GCP6mut corresponds to a deletion of GCP6 residues 329–341, while GCP5mut corresponds to a quadruple mutant of GCP5 R213A/R228G/L256E/V258E. Cells untransfected with any GCP-SBP-Myc constructs served as a negative control. Black triangle indicates location where blots were cropped for final figure generation. The experiment was performed three times with similar results. Right: partial alignment error plot for the AlphaFold prediction in Fig. 2 I. **(F)** Cartoon representation of AlphaFold 3 prediction of three copies each of GCP2 and GCP3 GRIP1 domains, together with the GCP6 belt and residues 191–252, colored by pLDDT (left) and subunit (right). (G) Segmented surface representation of the previously described helical element lining the lumenal face of GCP6, 2, and 3 in EMDB-11888 (Zimmermann et al., 2020). Map was postprocessed with EMready (He et al., 2023). The γ-TuRC subunits from the same study are shown in cartoon representation for reference. A zoomed in view of an unassigned helix contacting GCP6 is shown on the right and at a higher threshold.**(G)** Cartoon representation of AlphaFold 3 prediction of GRIP1 domains of GCP4, GCP5 (including NHD), GCP6, and GCP2, as well as MZT1 and the GRIP1 and GRIP2 domains of GCP3, colored by pLDDT (left) and subunit (right). The GCP5 insertion element that contacts the luminal face of GCP6 is indicated. **(H and I)** Segmented surface representation of the previously described helical element lining the luminal face of GCP6, GCP2, and GCP3 in EMDB-11888 (Zimmermann et al., 2020). Map was postprocessed with EMready (He et al., 2023). The γ-TuRC subunits from the same study are shown in cartoon representation for reference. A zoomed-in view of an unassigned helix contacting GCP6 is shown in I (left) and at a higher threshold. The right shows the GCP5 insertion (aa 567–608) modeled in this study in cartoon representation and fitted into the EMDB-11888 density map (Zimmermann et al., 2020). **(J)** GCP5 insertion modeled in this study (aa 567–608) is shown in cartoon representation in the rec-γ-TuRC density map. γ-TuRC position 12 corresponding to GCP6 is indicated in panels G, I, and J for reference. pLDDT, predicted local distance difference test. Source data are available for this figure: SourceData FS3.

The conclusions of the paper are not affected by these errors, and all discussion of the data presented remains correct. This error appears in print and any PDF downloaded prior to June 25, 2026. The authors apologize for any confusion this may have caused.

