# Supplementary material for: Structure of the microtubule-anchoring factor NEDD1 bound to the γ-tubulin ring complex
Source: J Cell Biol. 2025 May 21;224(8):e202410206. doi: 10.1083/jcb.202410206 (PMC12094035; doi:10.1083/jcb.202410206)
Supplement: Table S1 — shows the cryo-EM data collection. [file jcb_202410206_tables1.docx]

|  | **rec-γ-TuRC + CDK5RAP2** | | | | | |
| --- | --- | --- | --- | --- | --- | --- |
|  | Dataset 5 | Dataset 6 | Dataset 7 | Dataset 8 | Dataset 9 | Dataset 10 |
| Voltage (kV) | 300 | 300 | 300 | 300 | 300 | 300 |
| Magnification | 130,000X | 130,000X | 130,000X | 130,000X | 130,000X | 130,000X |
| Pixel size (Å/px) | 0.64 | 0.64 | 0.64 | 0.64 | 0.64 | 0.64 |
| Camera | K3 | K3 | K3 | K3 | K3 | K3 |
| GIF slit | 20 eV | 20 eV | 20 eV | 20 eV | 20 eV | 20 eV |
| Mode | CDS | CDS | CDS | CDS | CDS | CDS |
| Software | EPU | EPU | EPU | EPU | EPU | EPU |
| Spot size | 6 | 5 | 6 | 6 | 6 | 7 |
| Beam diameter (μm) | 0.82 | 0.78 | 0.81 | 0.86 | 0.80 | 0.57 |
| # of movies | 20,042 | 5,506 | 55,692 | 35,151 | 23,472 | 56,497 |
| # of frames | 50 | 50 | 50 | 46 | 46 | 40 |
| Exposure time (s) | 0.9 | 0.9 | 1 | 1 | 1 | 1 |
| Dose Rate (e^-^/px/s) | 31 | 27 | 24 | 23 | 25 | 26 |
| Total dose (e^-^/Å^2^) | 66 | 61 | 59 | 56 | 65 | 64 |
| Defocus range (μm) | -0.8/-2.4 | -0.8/-2.4 | -0.8/-2.4 | -0.8/-2.4 | -0.9/-2.4 | -1/-2.4 |
