# Supplementary material for: Structure of the microtubule-anchoring factor NEDD1 bound to the γ-tubulin ring complex
Source: J Cell Biol. 2025 May 21;224(8):e202410206. doi: 10.1083/jcb.202410206 (PMC12094035; doi:10.1083/jcb.202410206)
Supplement: Table S2 — shows the cryo-EM data processing statistics. [file jcb_202410206_tables2.docx]

|  | **rec-γ-TuRC**  **consensus map** | **rec-γ-TuRC + CDK5RAP2**  **consensus map** |
| --- | --- | --- |
| Processing pixel size (Å/pixel) | 1.32 | 1.41 |
| Box size (pixels) | 368 | 384 |
| Symmetry imposed | C1 | C1 |
| Number of particles | 266,675 | 71,778 |
| Resolution (Å)  (FSC 0.143 cutoff) | 4.7 (CS) | 6.8 (CS) |
| Resolution range (Å) (ResMap) | (3-11) | NC |
| Model building | Yes | Yes |
| Accession number | EMDB-53400 | EMDB-53399 |

*NC = Not calculated

*CS = CryoSPARC
