# Supplementary material for: Structure of the microtubule-anchoring factor NEDD1 bound to the γ-tubulin ring complex
Source: J Cell Biol. 2025 May 21;224(8):e202410206. doi: 10.1083/jcb.202410206 (PMC12094035; doi:10.1083/jcb.202410206)
Supplement: Table S3 — shows the model building and refinement statistics. [file jcb_202410206_tables3.docx]

|  | **rec-γ-TuRC** | **rec-γ-TuRC + CDK5RAP2** |
| --- | --- | --- |
| Cross-correlation | 0.43 | 0.38 |
| Number of chains | 45 | 48 |
| Number of residues | 17,575 | 17,794 |
| All atom clash score | 6.26 | 7.03 |
| Outliers (%) | 0.23 | 0.22 |
| Allowed (%) | 2.05 | 2.07 |
| Favoured (%) | 97.72 | 97.70 |
| Bond length (Å) | 0.002 | 0.002 |
| Bond angles (°) | 0.510 | 0.532 |
| Accession number | PDB: 9QVN | PDB: 9QVM |
