# Supplementary material for: Structure of the microtubule-anchoring factor NEDD1 bound to the γ-tubulin ring complex
Source: J Cell Biol. 2025 May 21;224(8):e202410206. doi: 10.1083/jcb.202410206 (PMC12094035; doi:10.1083/jcb.202410206)
Supplement: Table S4 — shows the (interface) predicted template modeling (ipTM & pTM) scores of AlphaFold predictions. [file jcb_202410206_tables4.docx]

| **Description** | **Protein name** | **UNIPROT accession** | **Residues** | **Copies** | **ipTM** | **pTM** |
| --- | --- | --- | --- | --- | --- | --- |
| NEDD1 pinwheel with MZT1:GCP3-NHD | NEDD1 | Q8NHV4 | 571-660 | 4 | 0.70 | 0.71 |
|  | MZT1 | Q08AG7 | 1-82 | 4 |  |  |
|  | GCP3 | Q96CW5 | 1-120 | 4 |  |  |
|  | | | | | | |
| NEDD1 pinwheel with MZT1:GCP2-NHD | NEDD1 | Q8NHV4 | 571-660 | 4 | 0.23 | 0.26 |
|  | MZT2 | Q6P582 | 1-158 | 4 |  |  |
|  | GCP2 | F2Z2B9 | 1-120 | 4 |  |  |
|  | | | | | | |
| *A. thaliana* NEDD1 with MZT1A & GCP3 | NEDD1 | B3H5K9 | 1-782 | 1 | 0.24 | 0.41 |
|  | MZT1A | Q9C9T3 | 1-67 | 1 |  |  |
|  | GCP3 | Q9FG37 | 1-838 | 1 |  |  |
|  | | | | | | |
| *A. thaliana* NEDD1 with MZT1A & GCP5B | NEDD1 | B3H5K9 | 1-782 | 1 | 0.19 | 0.40 |
|  | MZT1A | Q9C9T3 | 1-67 | 1 |  |  |
|  | GCP5 | A0A1P8AUV | 1-985 | 1 |  |  |
|  | | | | | | |
| NEDD1 pinwheel bound to GCP4/5/6 GRIP1 domains | GCP4 | Q9UGJ1 | 1-125 | 1 | 0.71 | 0.72 |
|  | GCP5 | Q96RT8 | 124-379 | 1 |  |  |
|  | GCP6 | AAI44489.1 | 282-497 | 1 |  |  |
|  | NEDD1 | Q8NHV4 | 582-660 | 4 |  |  |
|  | MZT1 | Q08AG7 | 1-82 | 4 |  |  |
|  | GCP3 | Q96CW5 | 1-120 | 4 |  |  |
|  | | | | | | |
| GCP6 belt extension | GCP2 | F2Z2B9 | 147-505 | 3 | 0.69 | 0.73 |
|  | GCP3 | Q96CW5 | 245-552 | 3 |  |  |
|  | GCP6 | AAI44489.1 | 117-240 | 1 |  |  |
|  | | | | | | |
| GCP5 insertion element | MZT1 | Q08AG7 | 1-82 | 2 | 0.53 | 0.58 |
|  | GCP3 | Q96CW5 | 1-907 | 1 |  |  |
|  | GCP2 | F2Z2B9 | 147-359 | 1 |  |  |
|  | GCP4 | Q9UGJ1 | 1-346 | 1 |  |  |
|  | GCP5 | Q96RT8 | 1-712 | 1 |  |  |
|  | GCP6 | AAI44489.1 | 195-605 | 1 |  |  |
|  | | | | | | |
| MZT1:GCP5-NHD as latch | γ-tubulin | P23258 | 1-457 | 1 | 0.86 | 0.85 |
|  | MZT1 | Q08AG7 | 1-82 | 1 |  |  |
|  | GCP5 | Q96RT8 | 1-135 | 1 |  |  |
|  | GCP3 | Q96CW5 | 245-907 | 1 |  |  |
|  | | | | | | |
| MZT1:GCP3-NHD as latch | γ-tubulin | P23258 | 1-457 | 1 | 0.76 | 0.79 |
|  | MZT1 | Q08AG7 | 1-82 | 1 |  |  |
|  | GCP3 | Q96CW5 | 1-120 | 1 |  |  |
|  | GCP3 | Q96CW5 | 245-907 | 1 |  |  |
