# Supplementary material for: Structure of the microtubule-anchoring factor NEDD1 bound to the γ-tubulin ring complex
Source: J Cell Biol. 2025 May 21;224(8):e202410206. doi: 10.1083/jcb.202410206 (PMC12094035; doi:10.1083/jcb.202410206)

SourceDataF2E-Myc

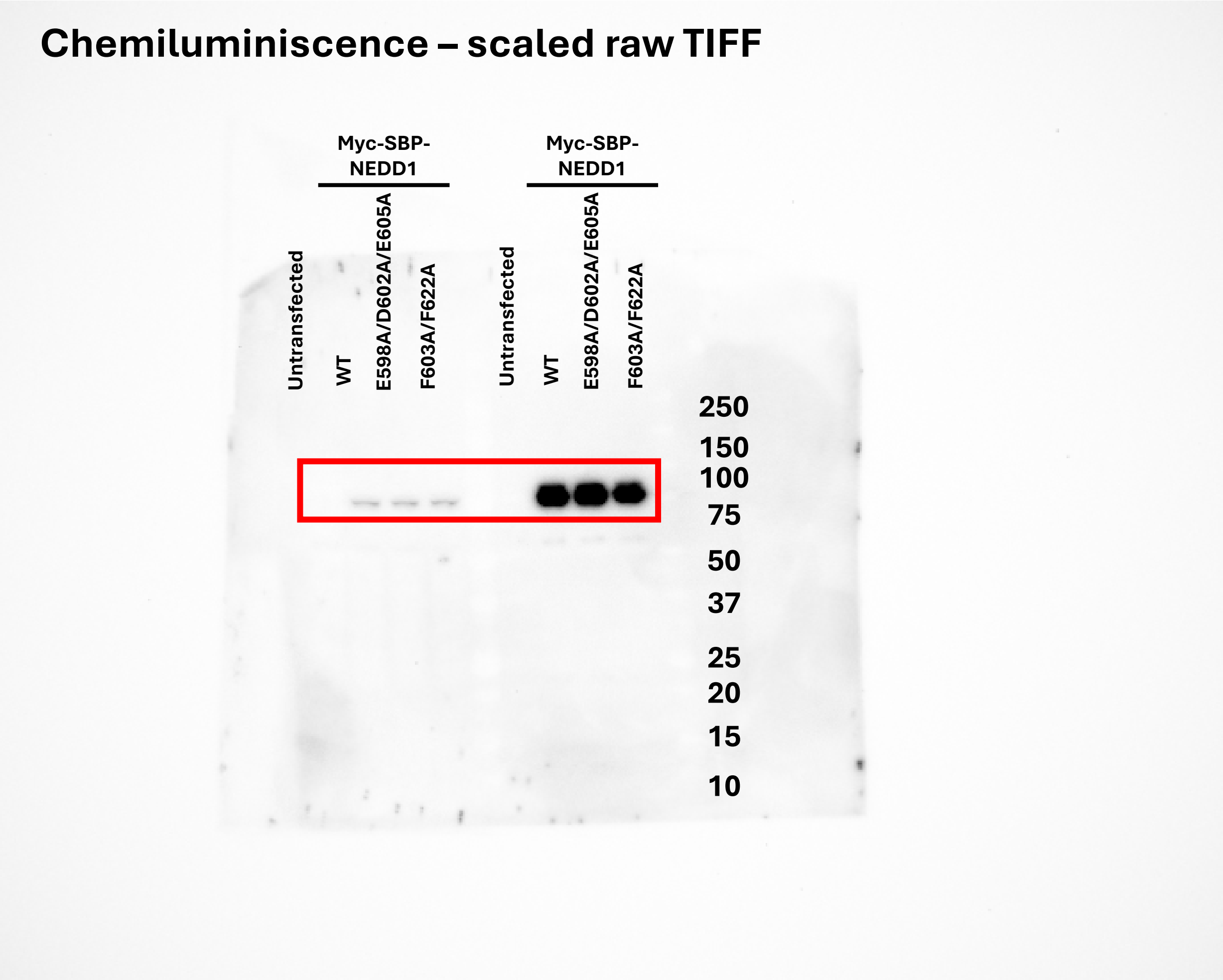

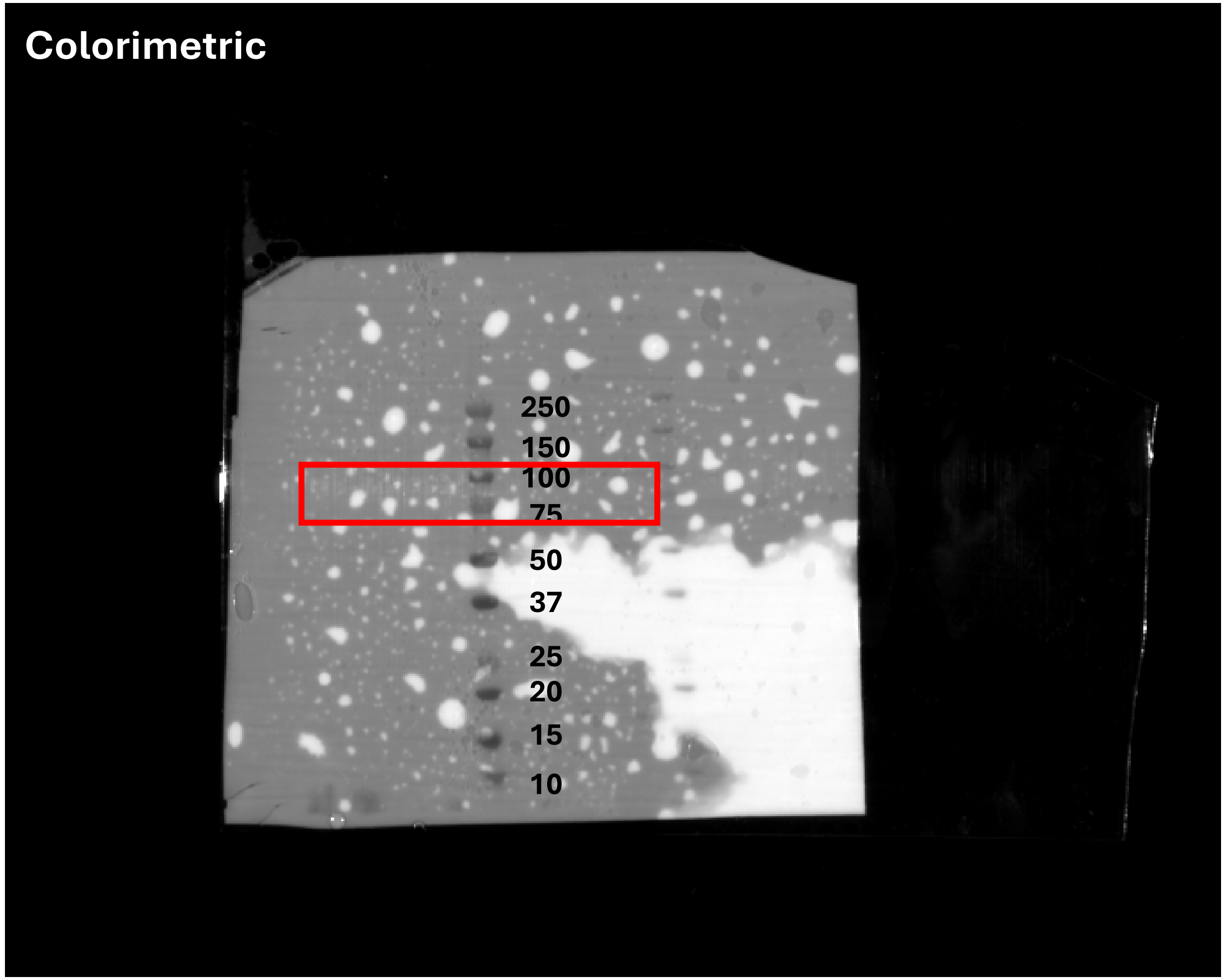

SourceDataF2E-Myc

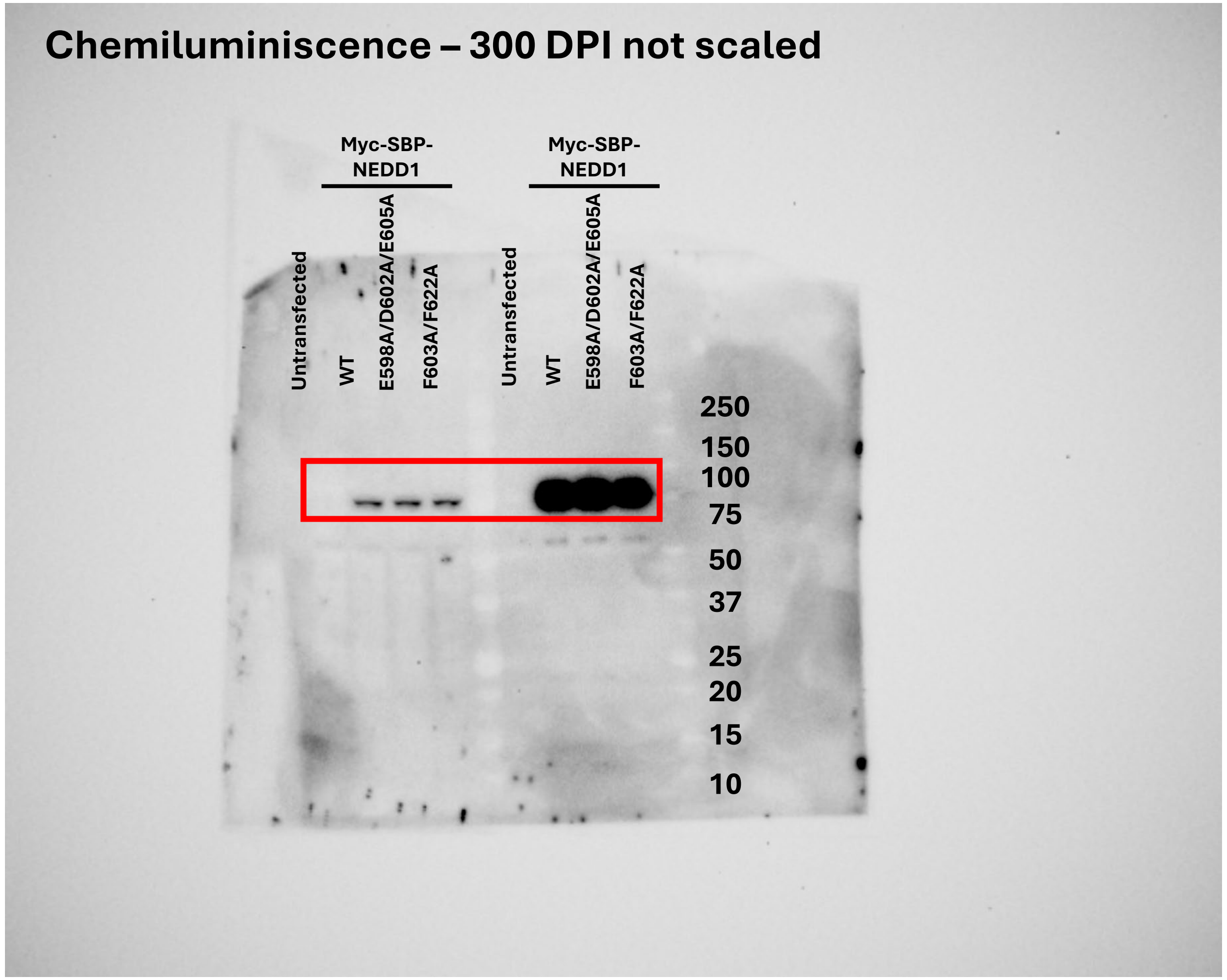

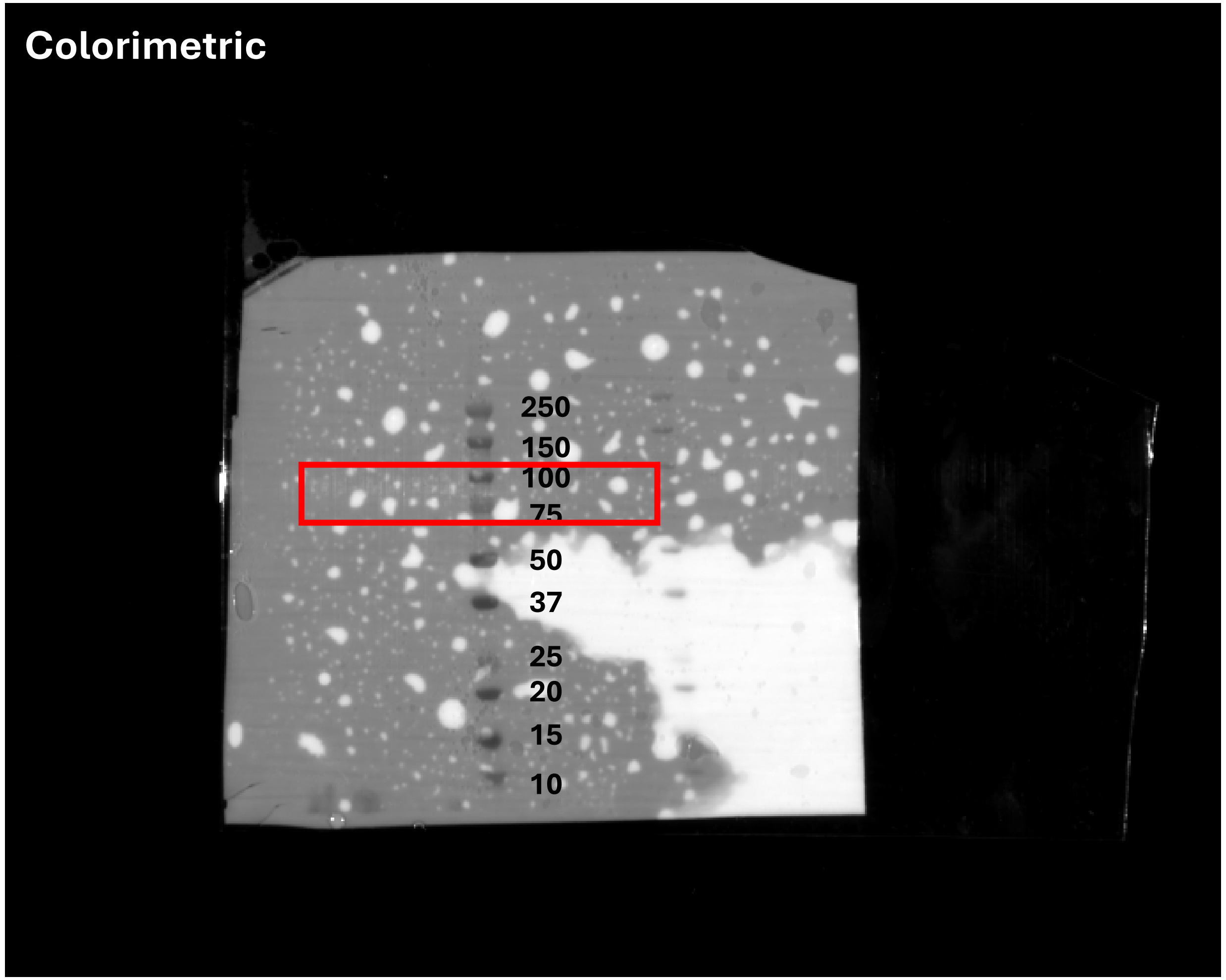

SourceDataF2E-γ-Tub

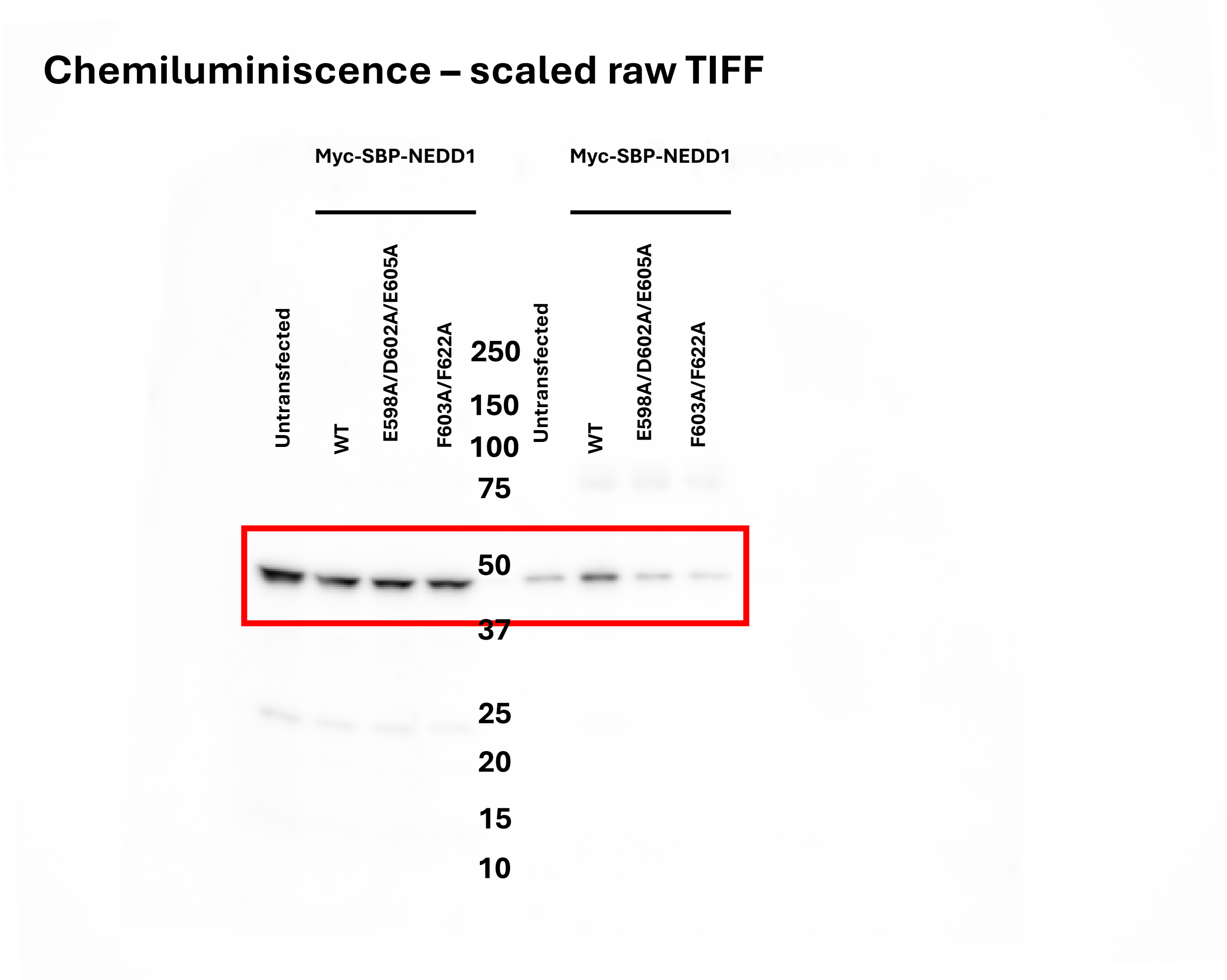

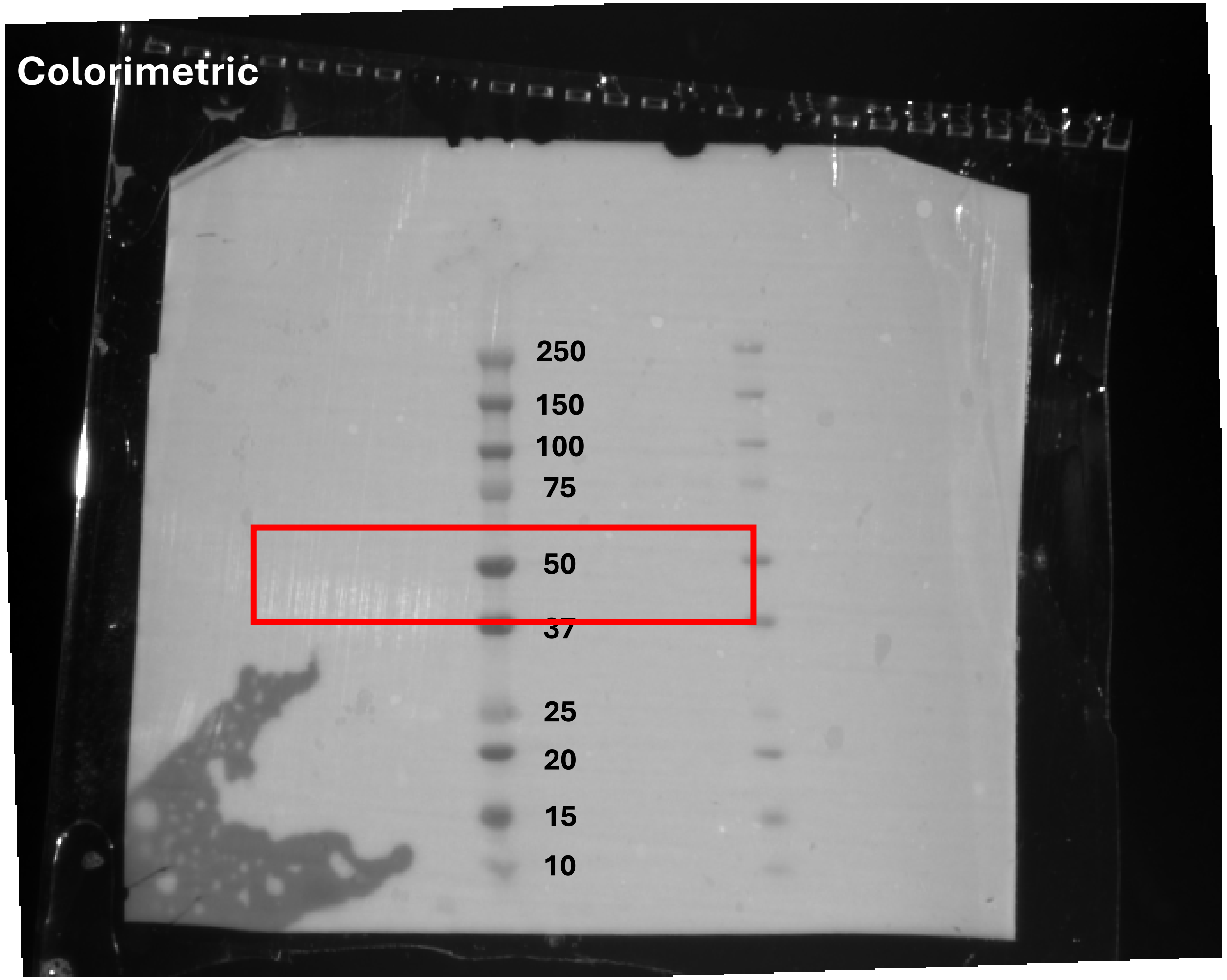

SourceDataF2E-γ-Tub

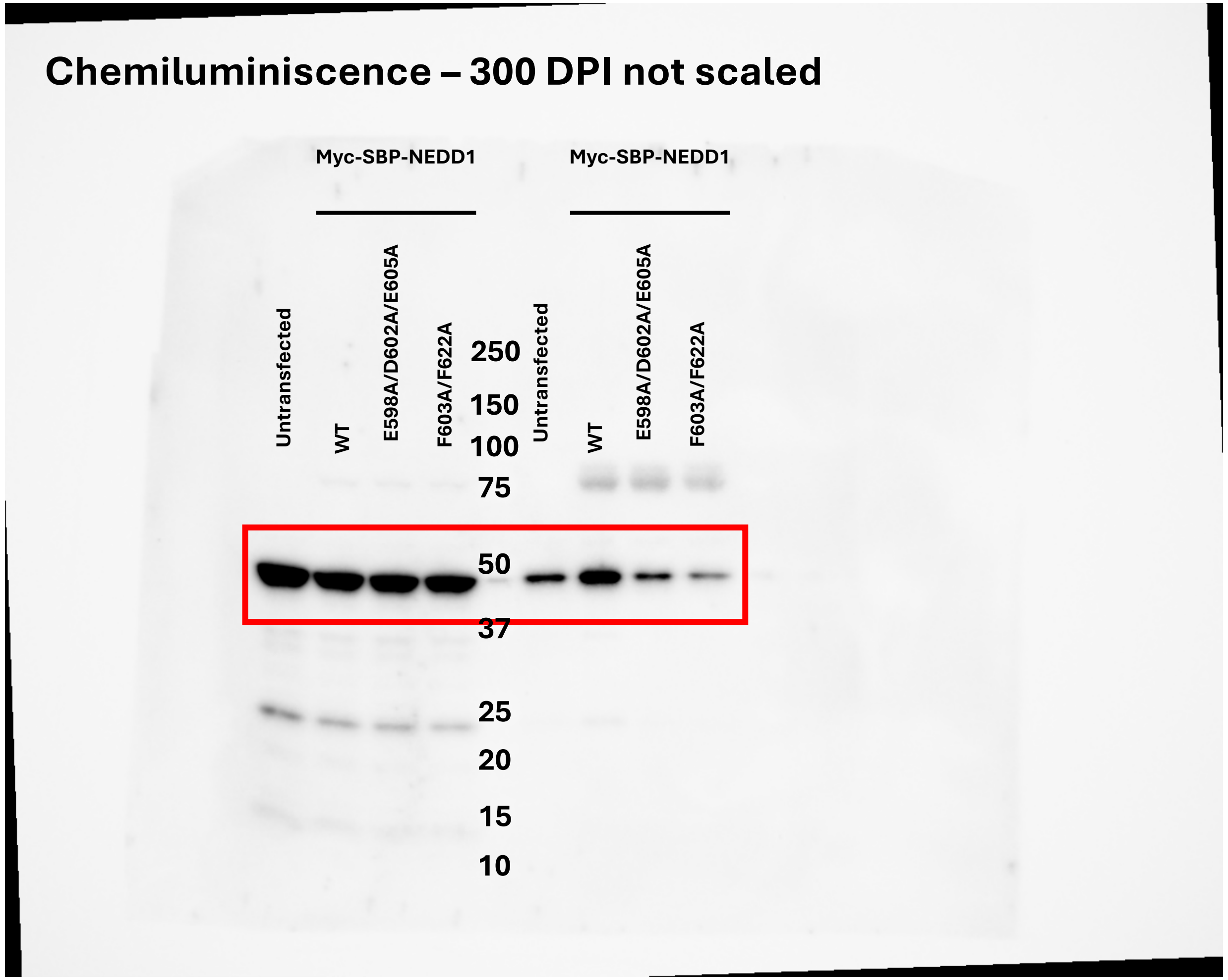

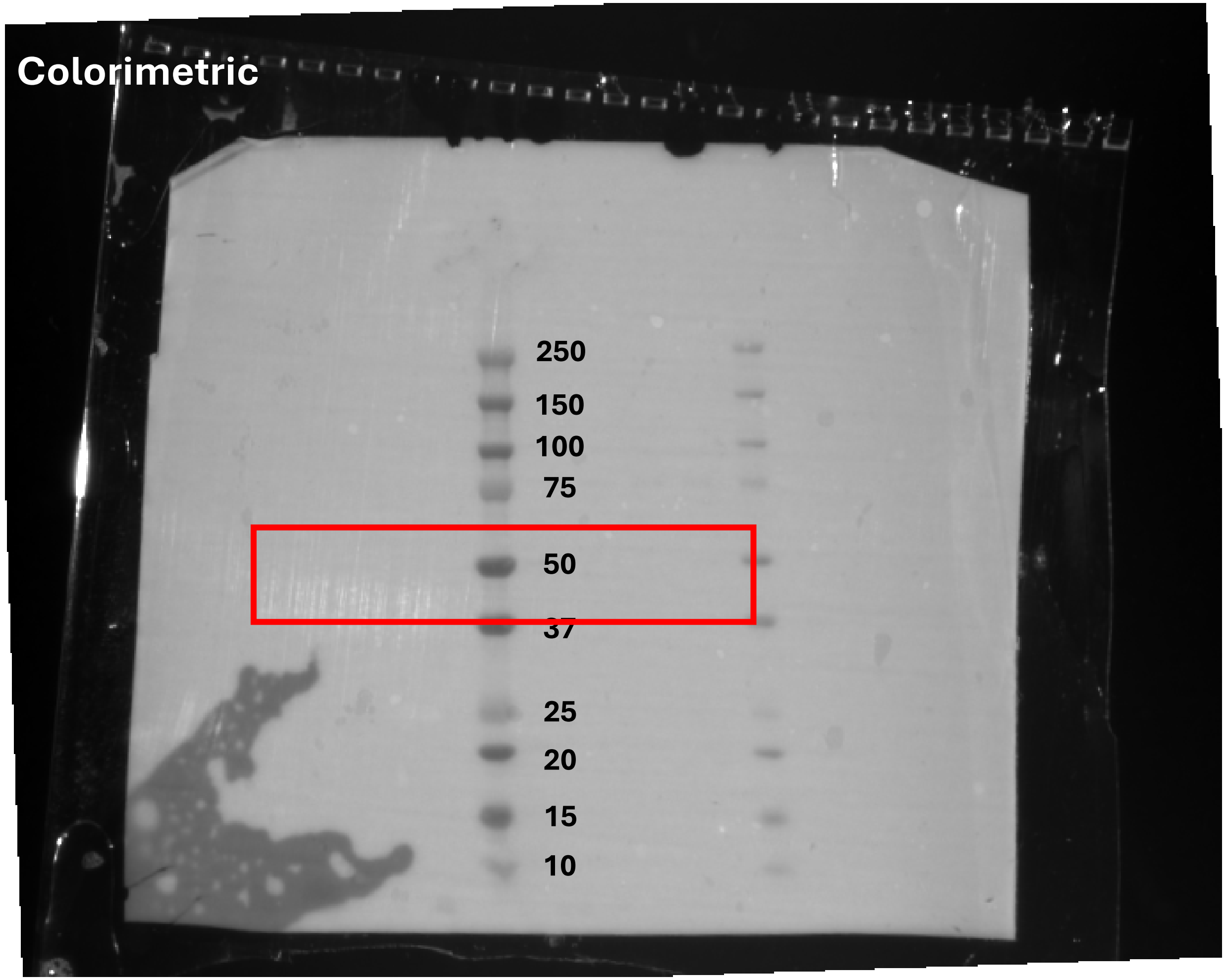

SourceDataF2G-Myc

Chemiluminiscence – scaled raw TIFF

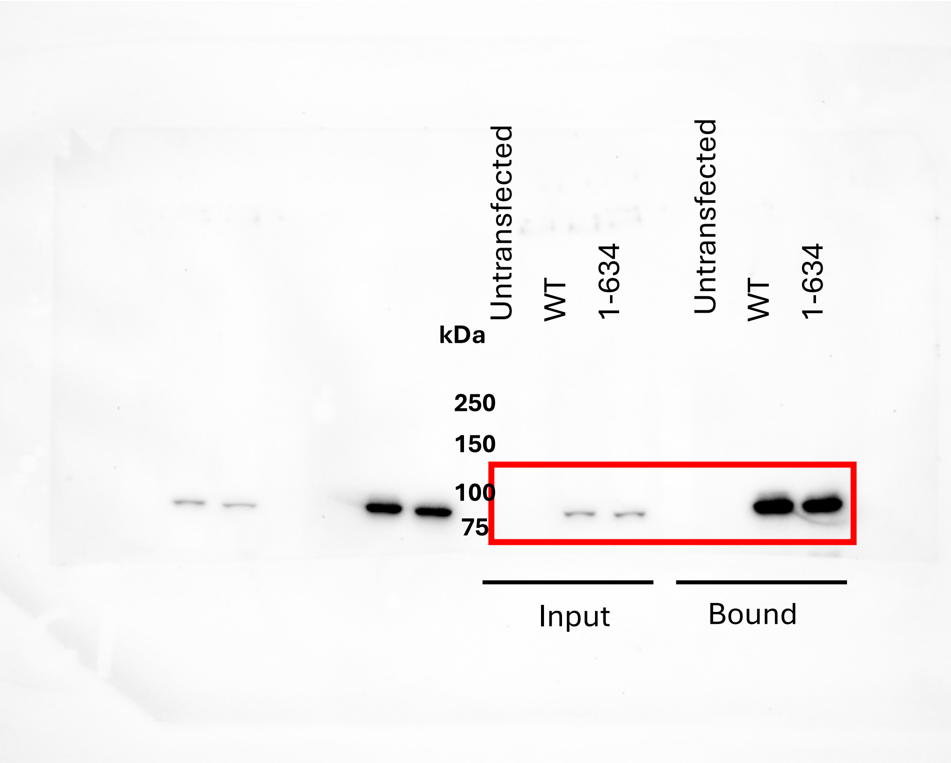

Colorimetric

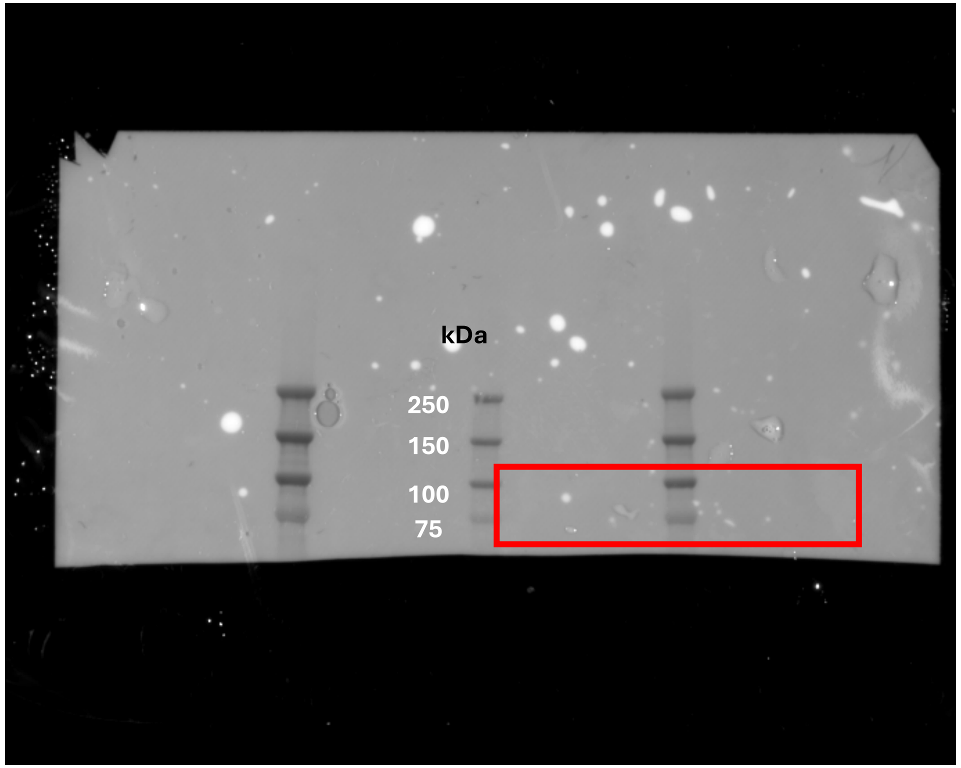

SourceDataF2G-Myc

Chemiluminiscence – 300 DPI not scaled

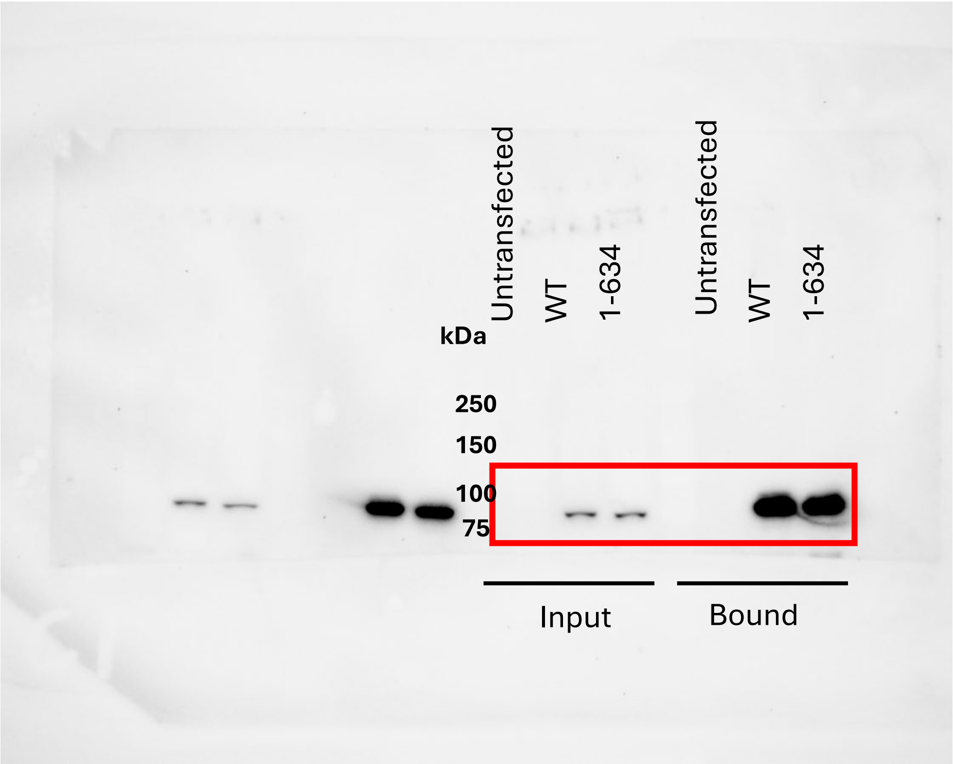

Colorimetric

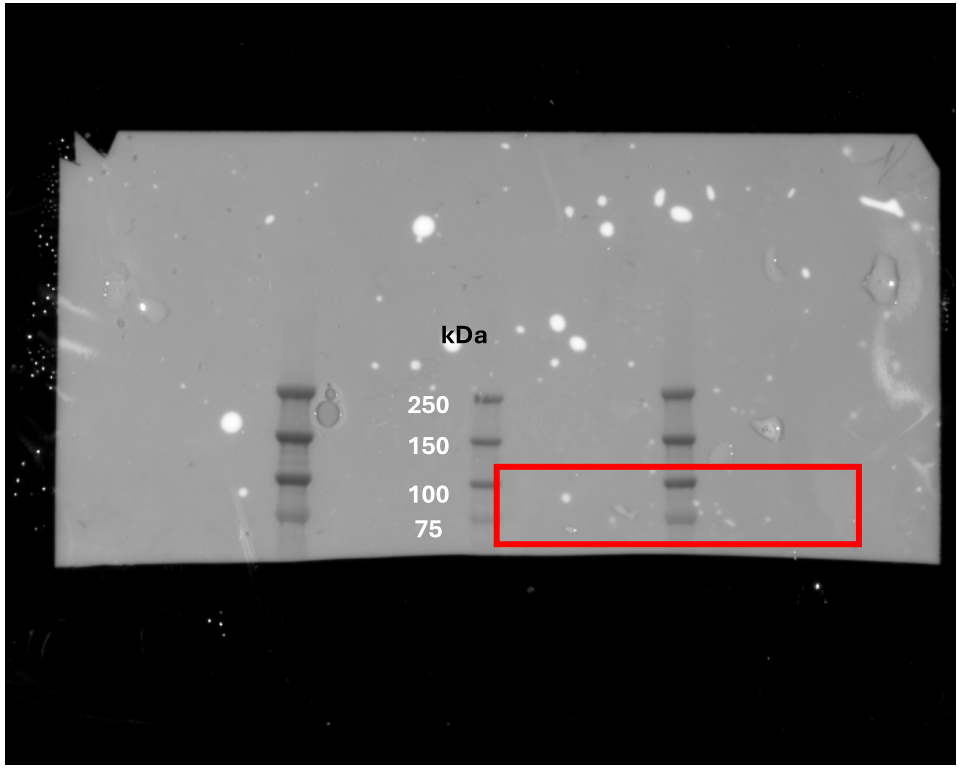

SourceDataF2G-γ-Tub

Chemiluminiscence – scaled raw TIFF

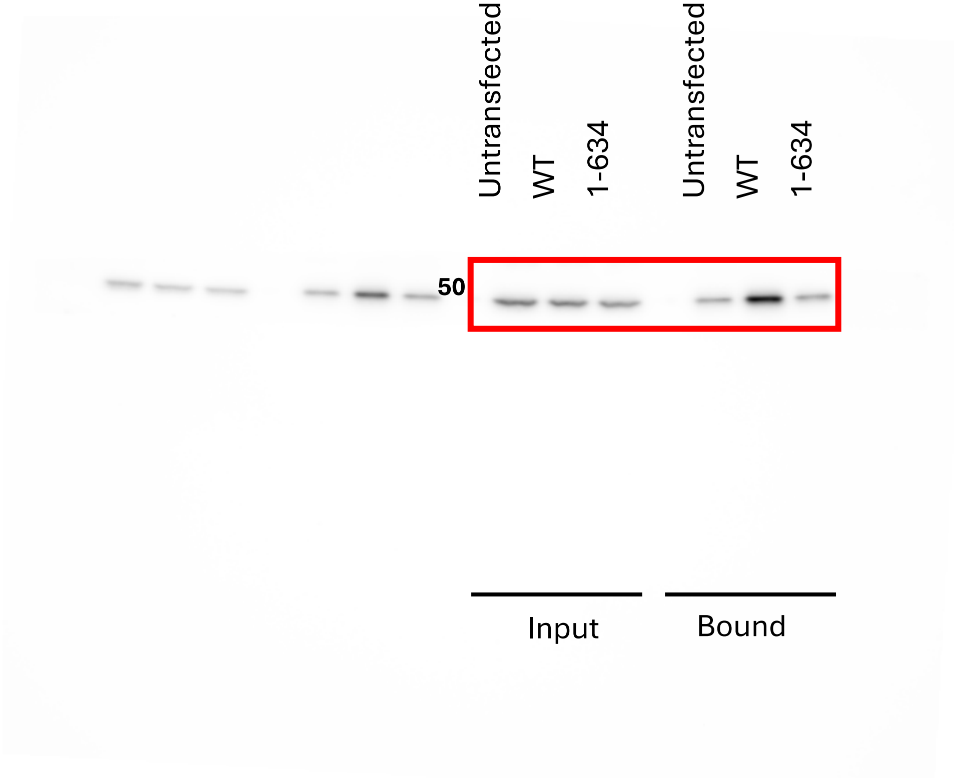

Colorimetric

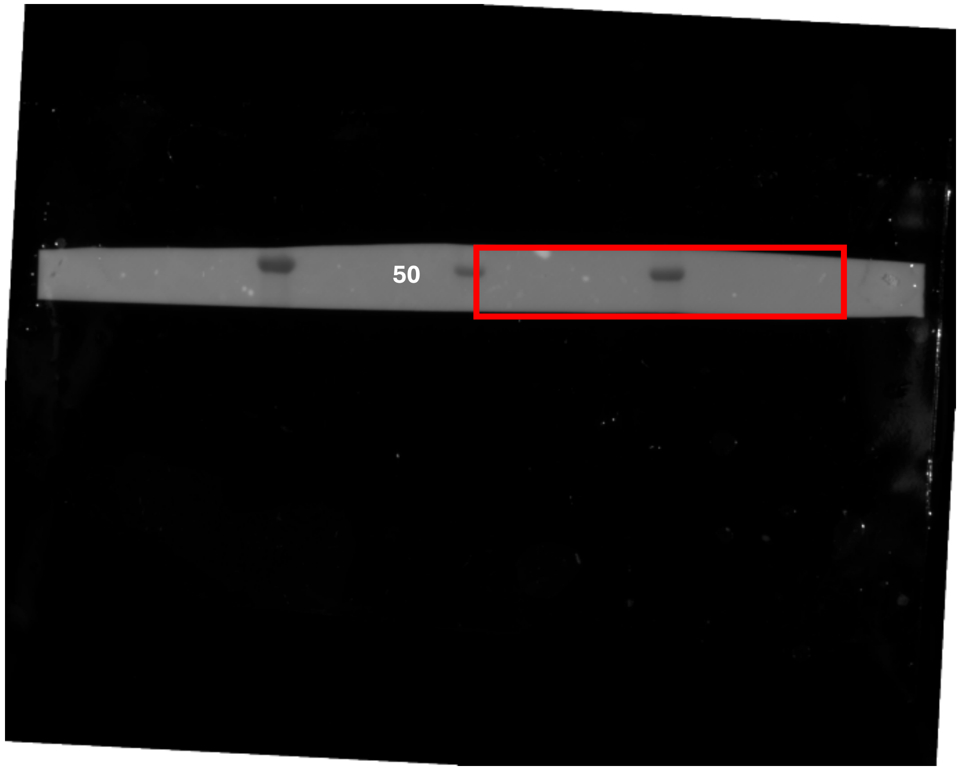

SourceDataF2G-γ-Tub

Chemiluminiscence - 300 DPI not scaled

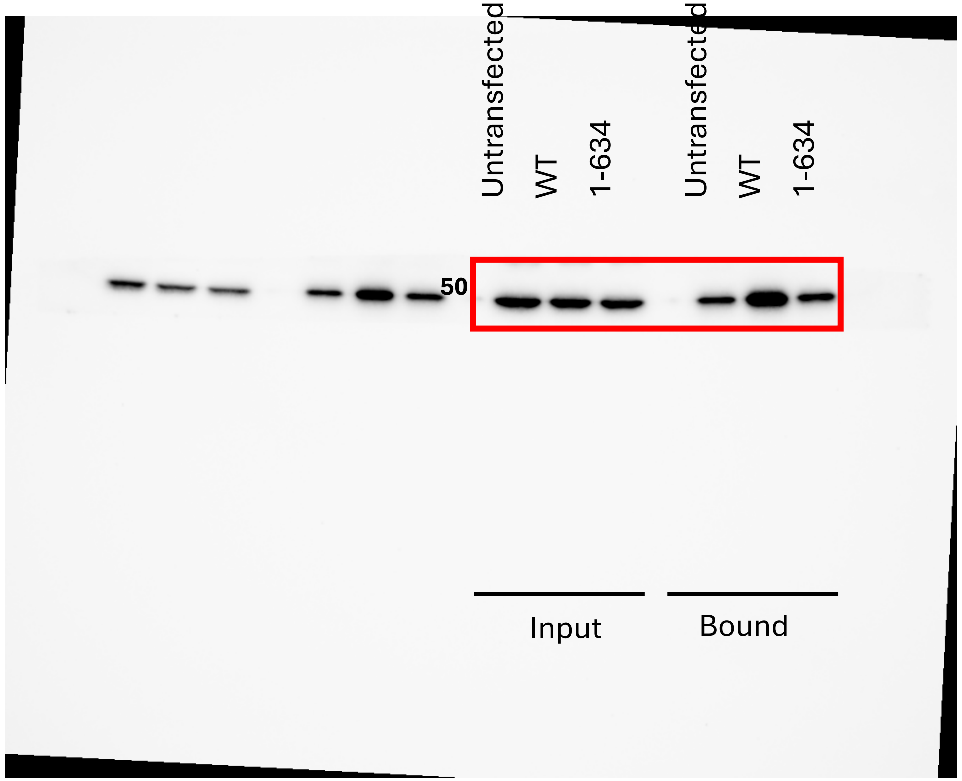

Colorimetric

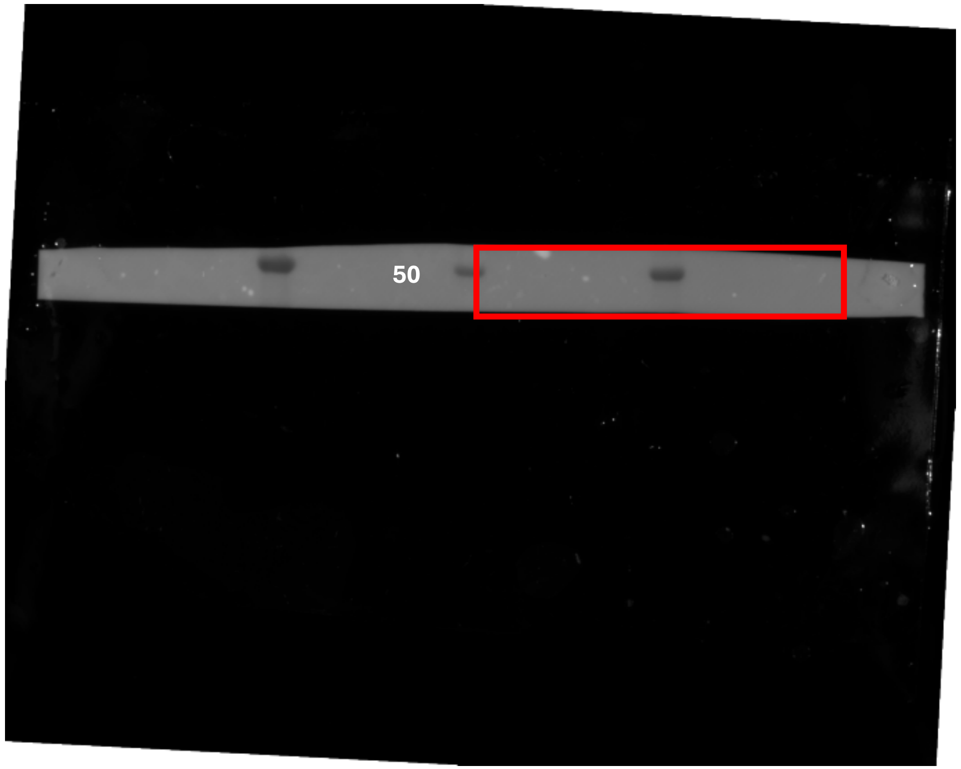

Supplement: SourceData F2 — is the source file for Fig. 2. [file jcb_202410206_sourcedataf2.pdf]
