# Supplementary material for: Structure of the microtubule-anchoring factor NEDD1 bound to the γ-tubulin ring complex
Source: J Cell Biol. 2025 May 21;224(8):e202410206. doi: 10.1083/jcb.202410206 (PMC12094035; doi:10.1083/jcb.202410206)

SourceDataSF3E-Myc

Chemiluminiscence – Raw TIFF scaled for input

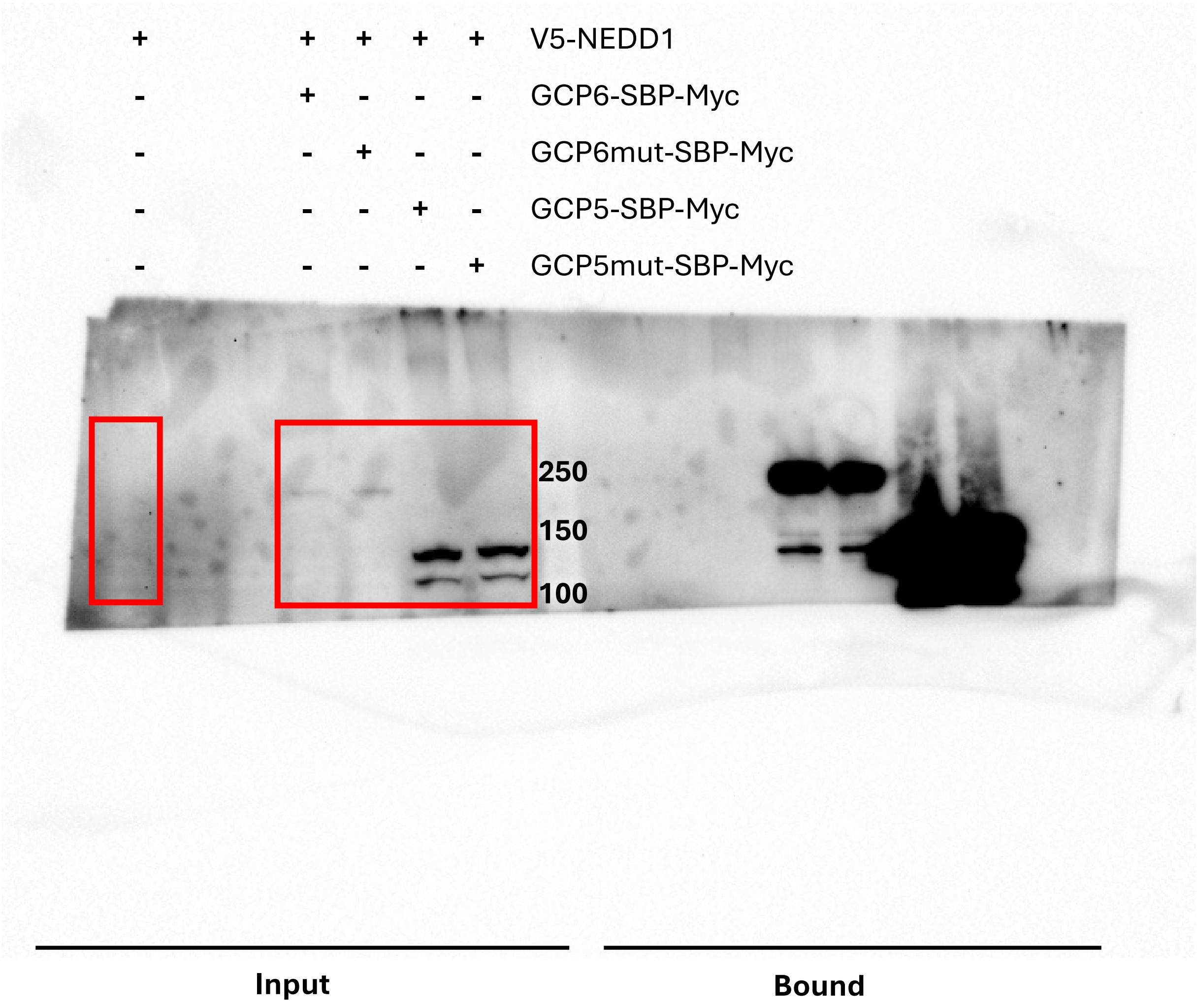

Colorimetric

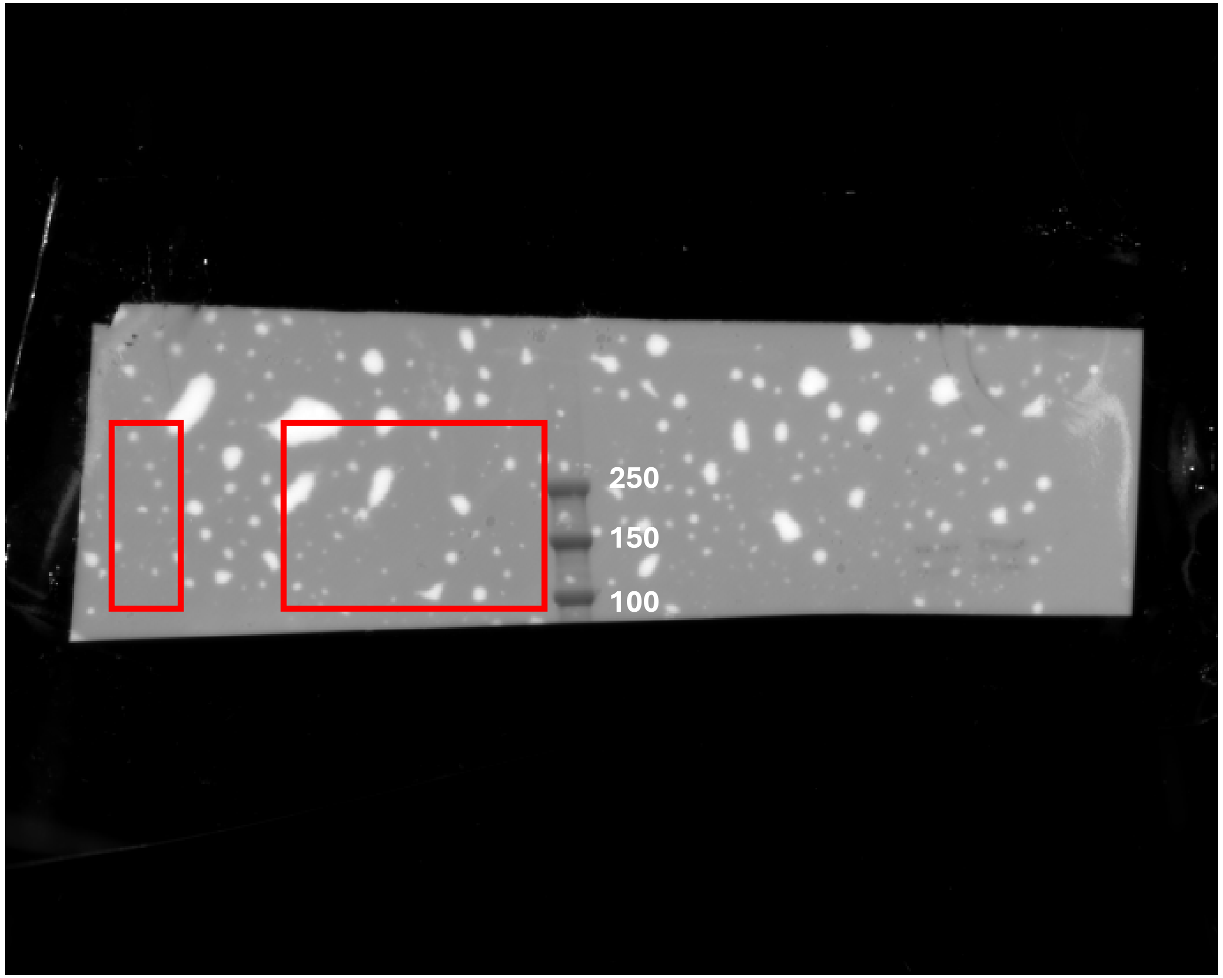

SourceDataSF3E-Myc

Chemiluminiscence – Raw TIFF scaled for Bound

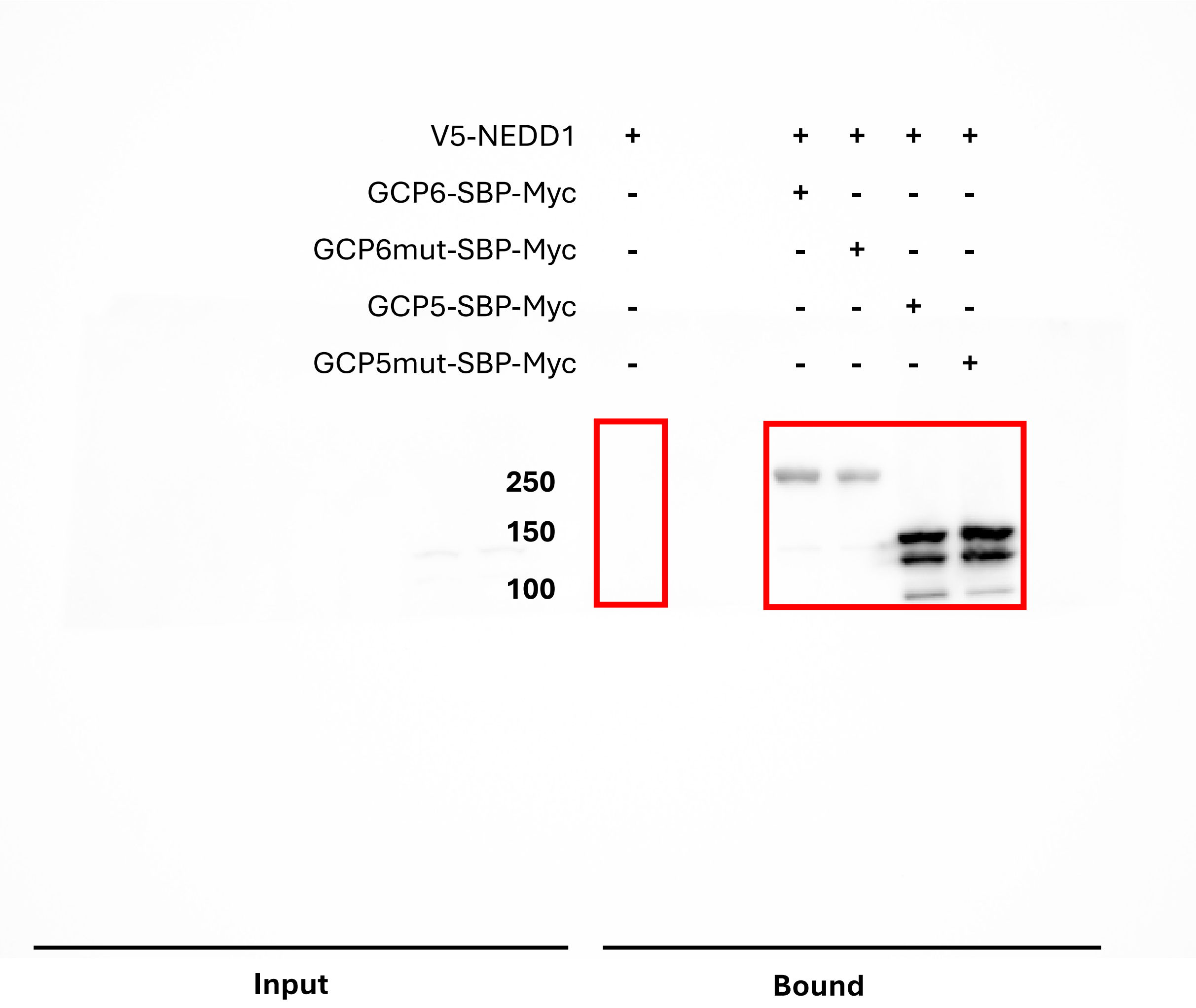

Colorimetric

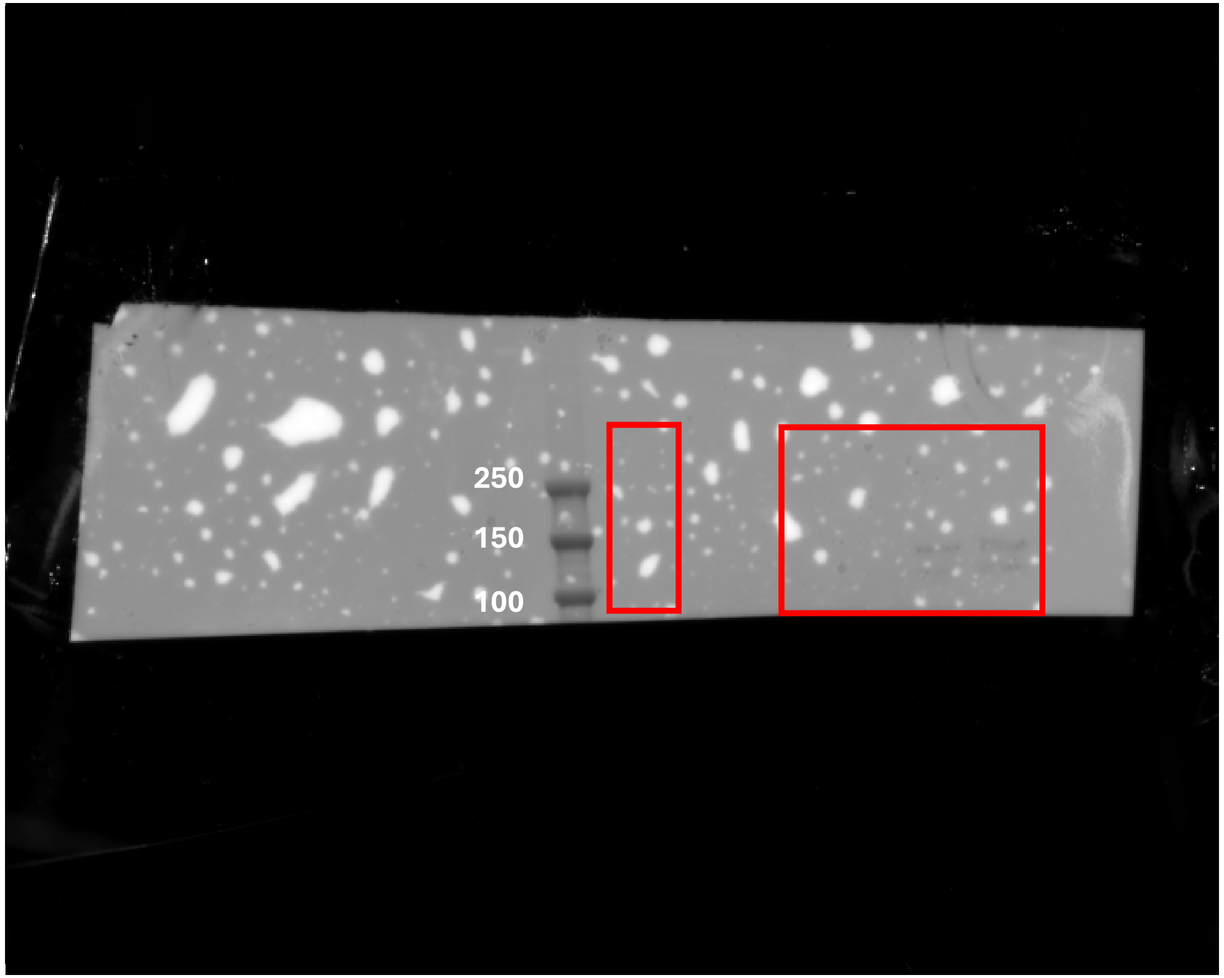

SourceDataSF3E-Myc

Chemiluminiscence – 300 DPI not scaled

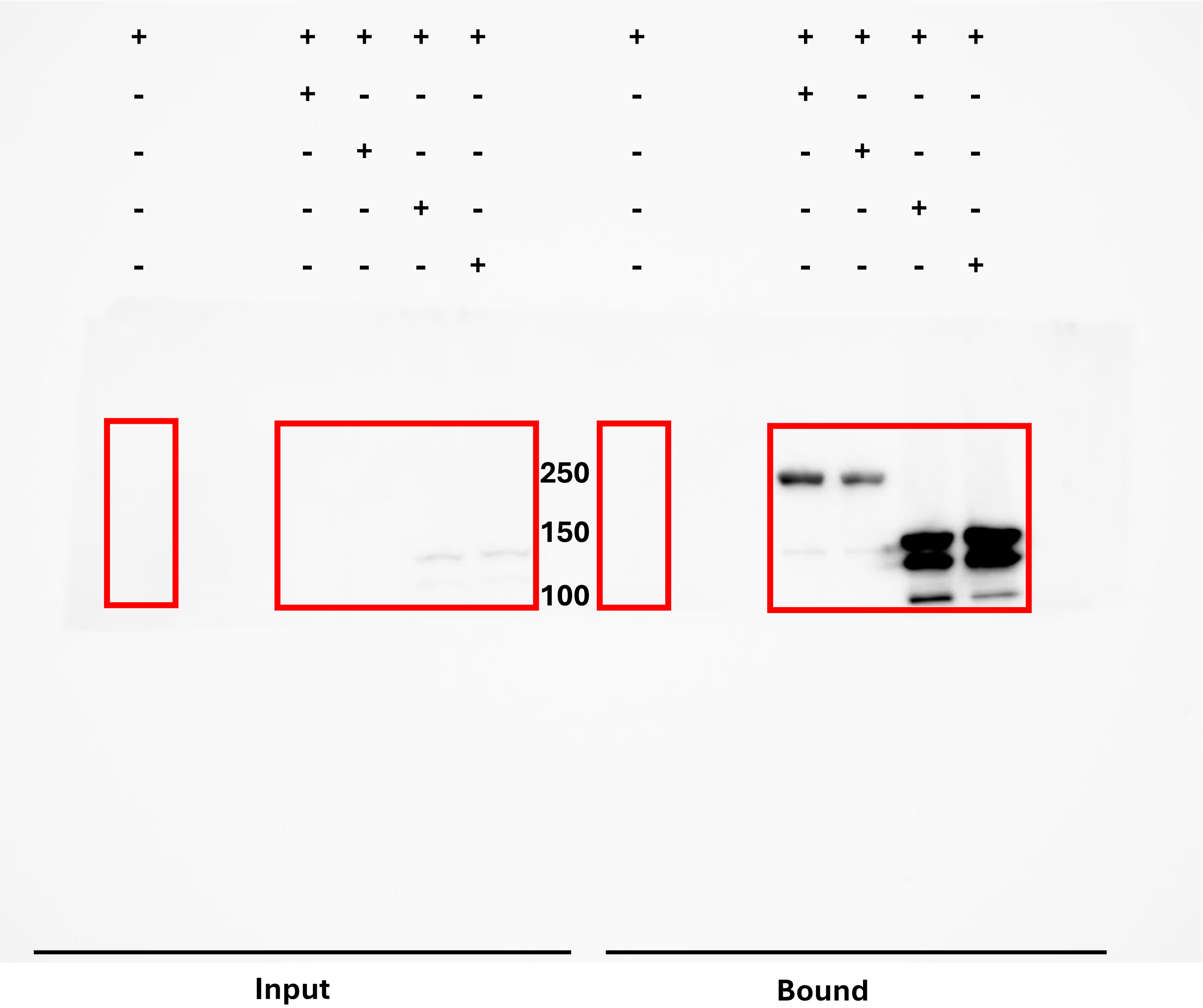

Colorimetric

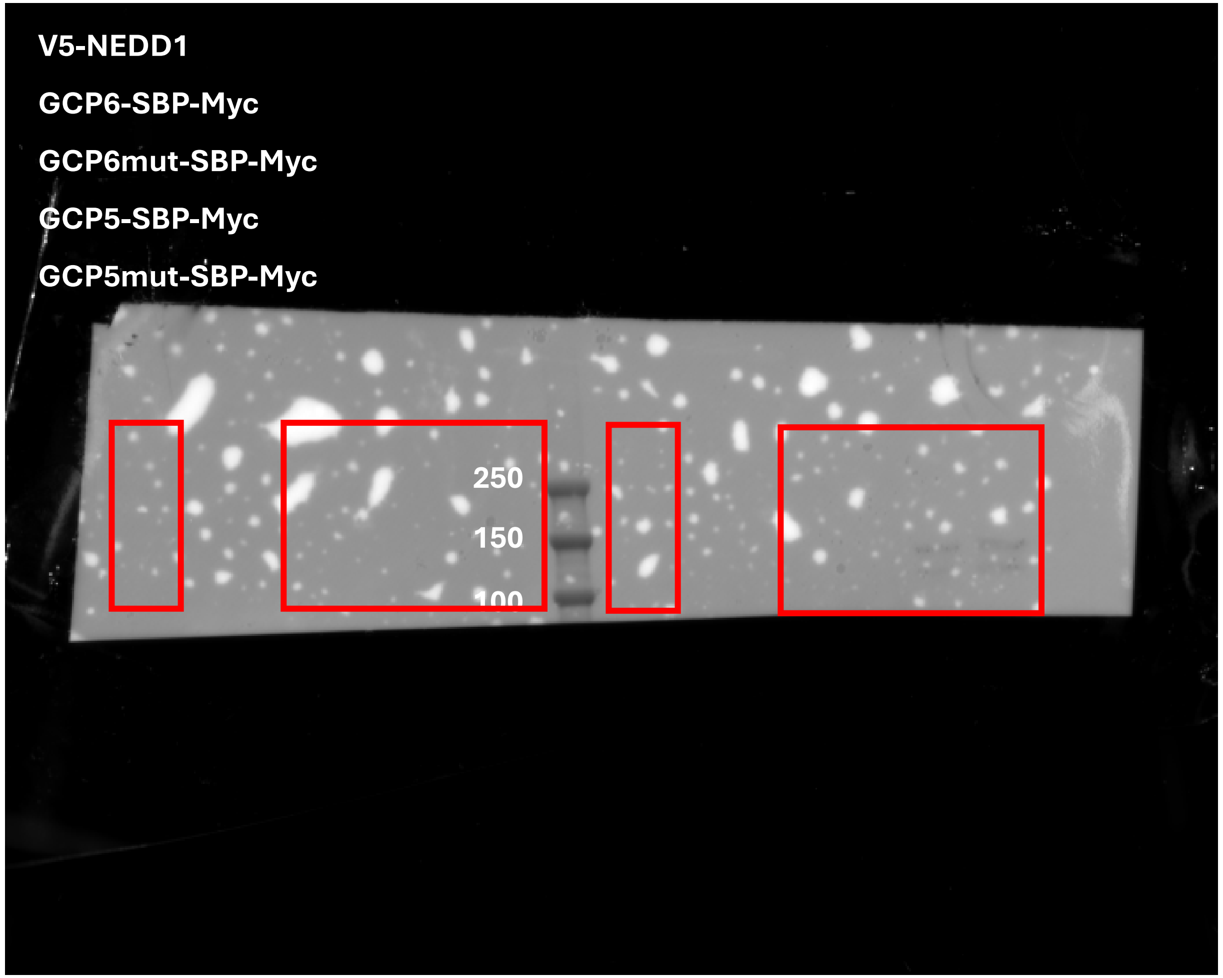

SourceDataSF3E-V5

Chemiluminiscence – Raw TIFF scaled

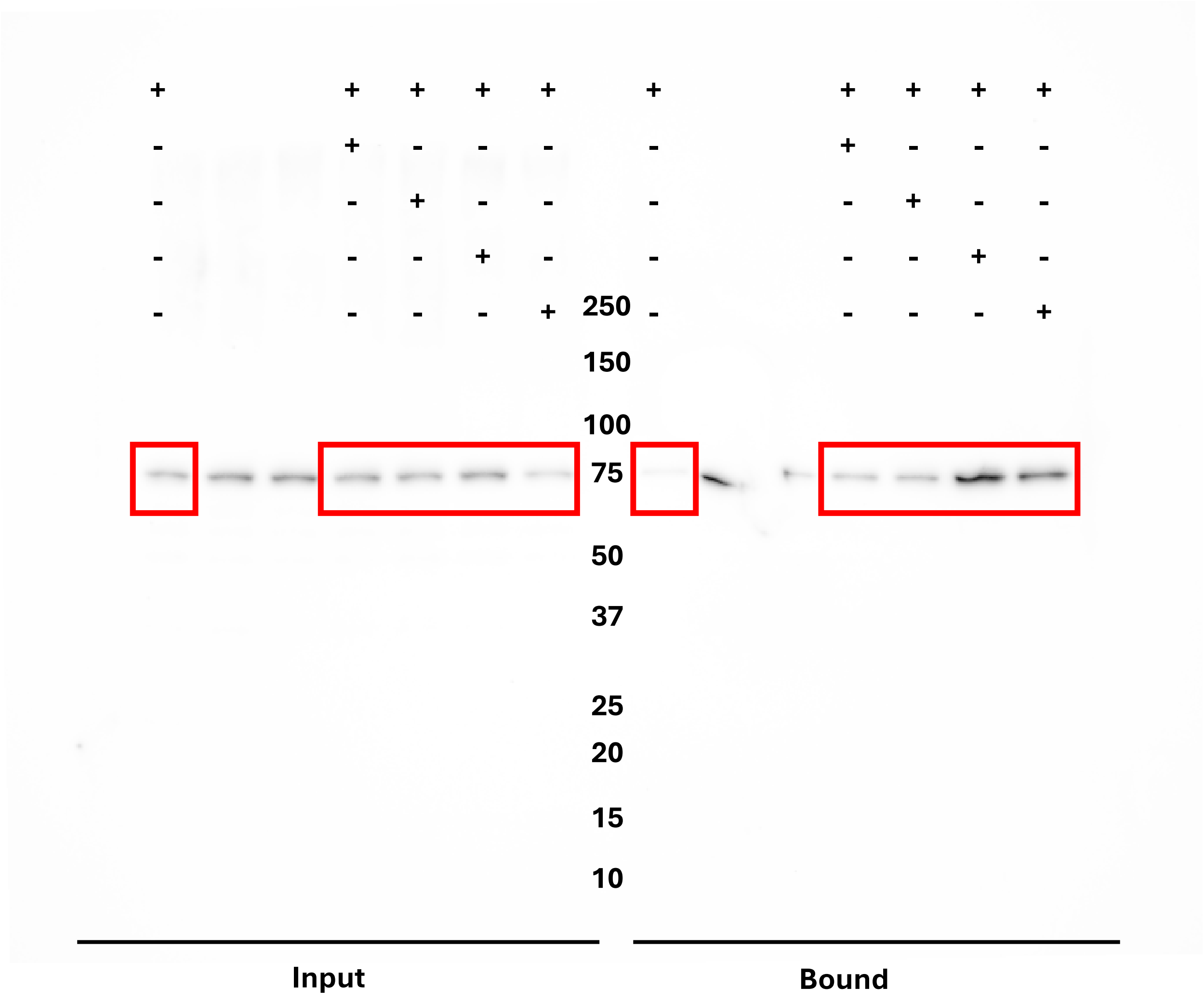

Colorimetric

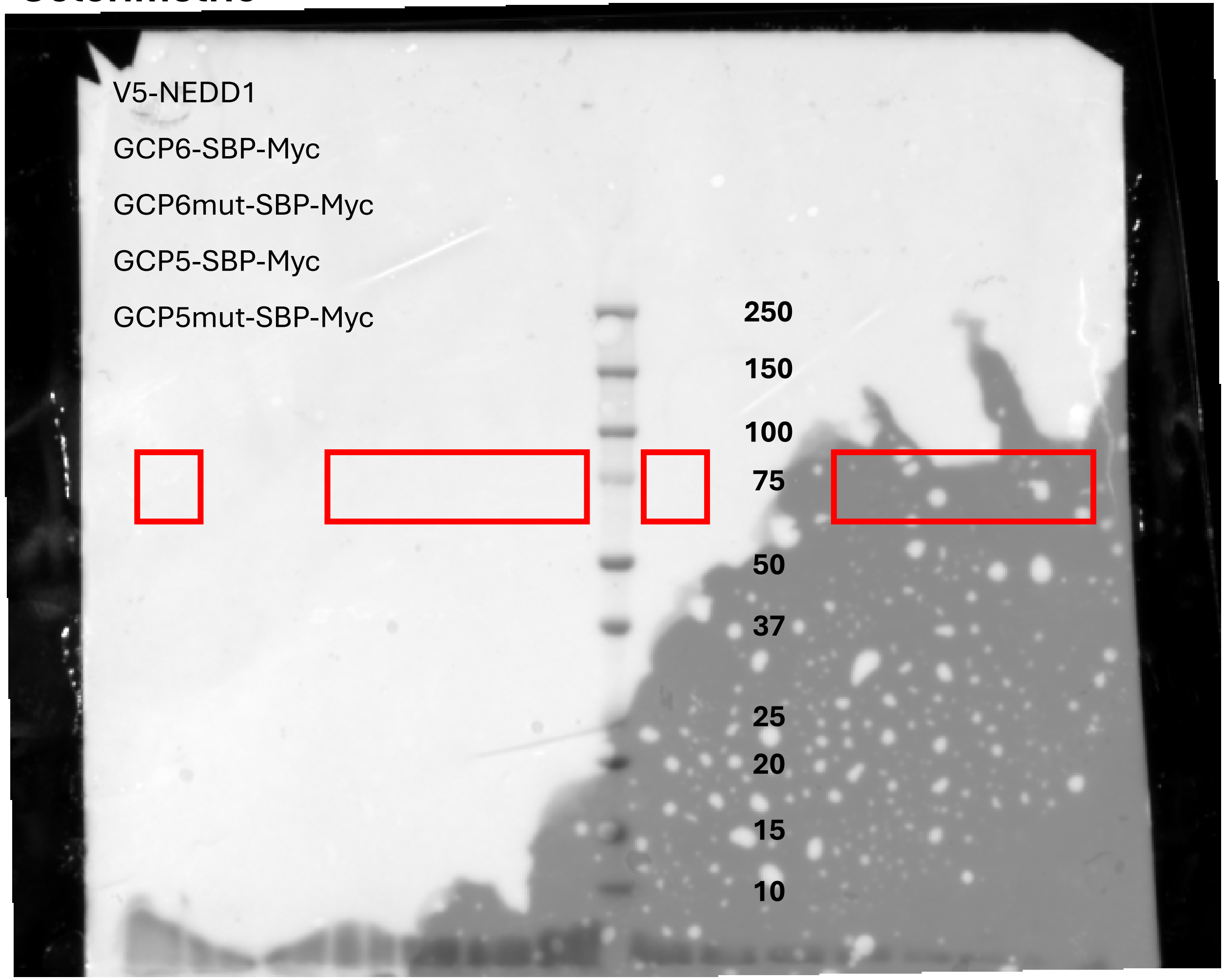

SourceDataSF3E-V5

Chemiluminiscence – 300 DPI not scaled

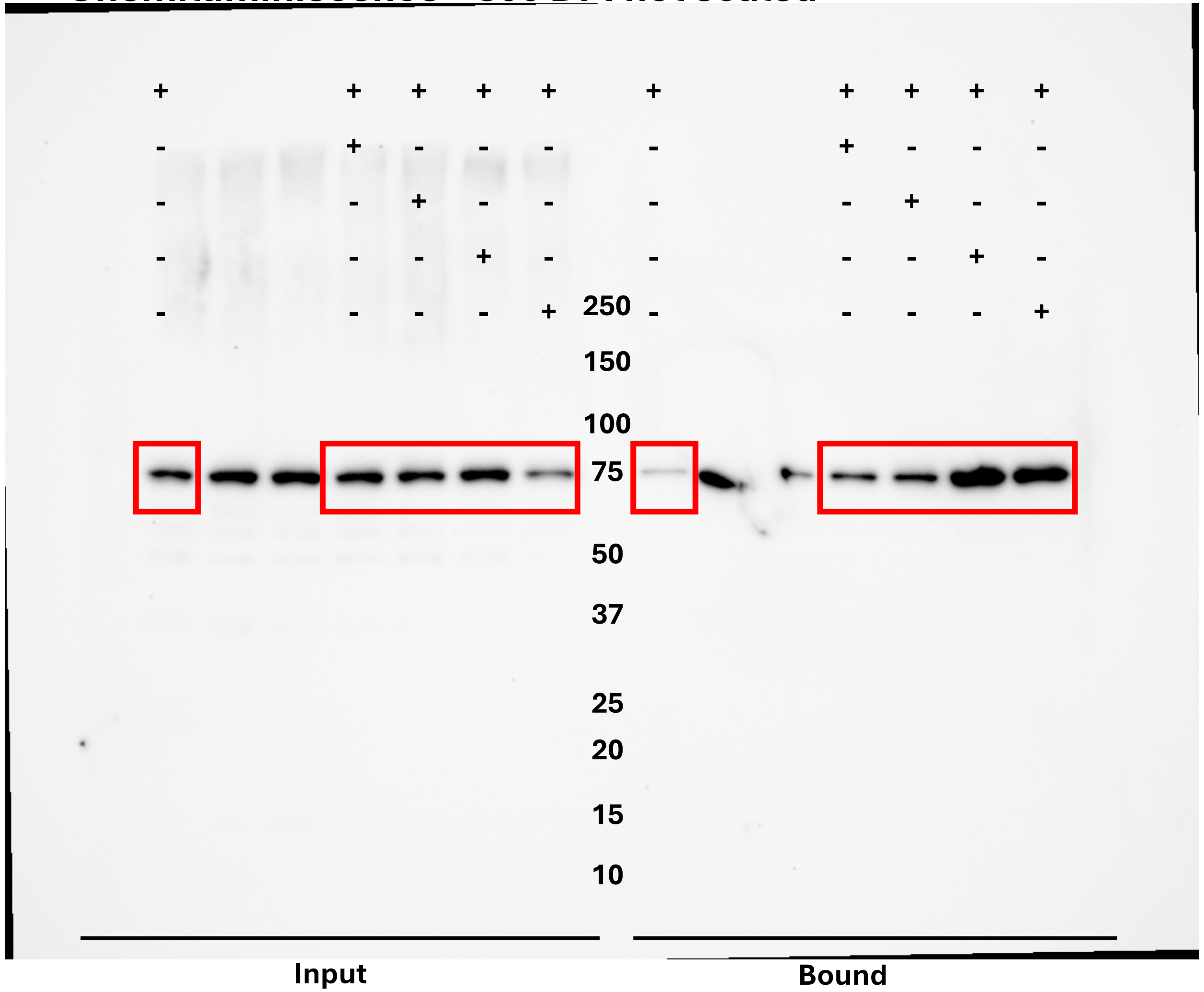

Colorimetric

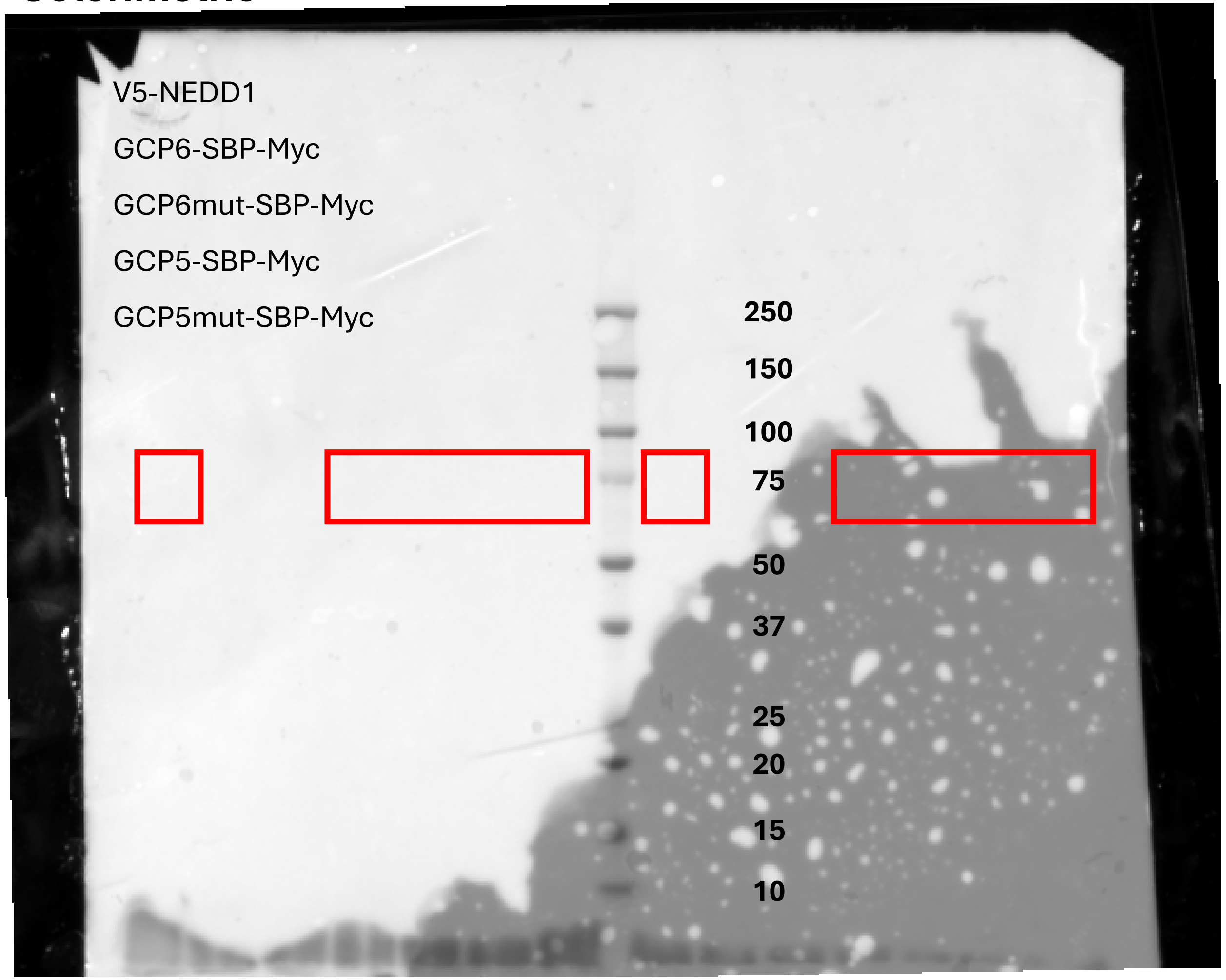

Supplement: SourceData FS3 — is the source file for Fig. S3. [file jcb_202410206_sourcedatafs3.pdf]
